# Effect of Peanut Consumption on Cardiovascular Risk Factors: A Randomized Clinical Trial and Meta-Analysis

**DOI:** 10.3389/fnut.2022.853378

**Published:** 2022-04-01

**Authors:** Isabella Parilli-Moser, Sara Hurtado-Barroso, Marta Guasch-Ferré, Rosa M. Lamuela-Raventós

**Affiliations:** ^1^Department of Nutrition, Food Science and Gastronomy, School of Pharmacy and Food Sciences XIA, Institute of Nutrition and Food Safety (INSA-UB), University of Barcelona, Barcelona, Spain; ^2^Spanish Biomedical Research Centre in Physiopathology of Obesity and Nutrition (CIBERobn), Carlos III Health Institute (ISCIII), Madrid, Spain; ^3^Department of Nutrition, Harvard T. H. Chan School of Public Health, Boston, MA, United States; ^4^Channing Division of Network Medicine, Department of Medicine, Brigham and Women's Hospital and Harvard Medical School, Boston, MA, United States

**Keywords:** ARISTOTLE, lipid profile, nuts, cardiometabolic risk, health, triglycerides

## Abstract

Although numerous studies have reported the protective effect of nut consumption on cardiovascular risk, evidence for the role of peanuts in maintaining cardiometabolic health is inconclusive. Presented here are the results from the ARISTOTLE study, a parallel randomized controlled trial evaluating the impact of regular peanut intake on anthropometric, biochemical, and clinical measurements. The 63 healthy subjects that completed the study consumed their habitual diet plus either: a) 25 g/day of skin roasted peanuts (SRP, *n* = 21), b) two tablespoons (32 g)/day of peanut butter (PB, *n* = 23) or c) two tablespoons (32 g)/day of a control butter based on peanut oil (CB, *n* = 19) for 6 months. In addition, a meta-analysis of clinical trials, including data from the ARISTOTLE study, was carried out to update the evidence for the effects of consuming peanuts, including high-oleic peanuts, and peanut butter on healthy subjects and those at high cardiometabolic risk. After a systematic search on PubMed, Web of Science, Cochrane Library and Scopus databases up to July 2021, 11 studies were found to meet the eligibility criteria. In the ARISTOTLE study, lower total cholesterol/HDL-cholesterol and LDL-cholesterol/HDL-cholesterol ratios were found in the SRP group compared to the CB group (*p* = 0.019 and *p* = 0.008). The meta-analysis of clinical trials revealed that peanut consumption is associated with a decrease in triglycerides (MD: −0.13; 95% CI, −0.20 to −0.07; *p* < 0.0001) and that healthy consumers had lower total cholesterol and LDL-cholesterol/HDL-cholesterol ratios compared to the control groups (MD: −0.40; 95% CI, −0.71 to −0.09; *p* = 0.01 and MD: −0.19; 95% CI, −0.36 to −0.01; *p* = 0.03, respectively). However, individuals at high cardiometabolic risk experienced an increase in body weight after the peanut interventions (MD: 0.97; 95% CI, 0.54 to 1.41; *p* < 0.0001), although not in body fat or body mass index. According to the dose-response analyses, body weight increased slightly with higher doses of peanuts. In conclusion, a regular consumption of peanuts seems to modulate lipid metabolism, reducing triglyceride blood levels.

**Systematic Review Registration:**

https://osf.io/jx34y/, identifier: 10.17605/OSF.IO/MK35Y.

## Introduction

Peanuts are the most consumed nuts worldwide. In 2018, the global consumption of peanuts increased to ~42.6 million metric tons, which is 10-fold higher than that of tree nuts (1). The sustainability and low cost of peanut production makes them more affordable than other nuts (2). Numerous studies indicate that peanut consumption may have a positive effect on cardiometabolic biomarkers, and reduce the risk of total cardiovascular and coronary heart disease (3–8). Peanuts are a rich source of nutritious and bioactive components, including protein, fiber, folate, niacin, magnesium, selenium, arginine, α-tocopherol, manganese, monounsaturated fatty acids, and phytochemicals such as polyphenols and phytosterols, which have a protective affect against cardiovascular disease (9–11).

Studies evaluating the effects of peanut consumption on cardiovascular risk factors have reported conflicting results, possibly due to differences in sample size, intervention products or study duration. Therefore, our aim was i) to evaluate the health impact of peanut products in a 6-month parallel randomized clinical trial, which was carried out between November 2019 and June 2020, and ii) to update the existing evidence for the effects of consuming peanuts, including high-oleic peanuts, and peanut butter on cardiometabolic risk by conducting a meta-analysis of controlled trials.

## Methods

### Study Design

The ARISTOTLE study is a three-arm parallel-group randomized controlled trial (NCT04324749), approved by the Ethics Committee of Clinical Investigation of the University of Barcelona (Barcelona, Spain) and conducted according to the principles of the Declaration of Helsinki. The 63 healthy volunteers, aged between 18 and 33 years, who completed the ARISTOTLE study were recruited from the Food and Nutrition Torribera Campus of the University of Barcelona and surrounding area and signed an informed consent prior to the start of the trial. The exclusion criteria were as follows: a history of chronic diseases (cardiovascular diseases, cancer, diabetes, and others), peanut allergy or intolerance, body mass index (BMI) over 25 kg/m2, active smoking, high alcohol consumption and other toxic habits.

At baseline, participants were randomized to three intervention groups, consuming either a) 25 g/day of skin roasted peanuts (SRP) or b) two tablespoons (32 g)/day of peanut butter (PB) or c) two tablespoons (32 g)/day of a control butter based on peanut oil, free of fiber and polyphenols (CB). The intervention period was 6 months, but due to the COVID-19 pandemic, in some cases it was extended to 7 months. The volunteers were supplied with the three intervention products and requested to follow a peanut-free diet for 2 weeks before the start of the study. During the intervention, they followed their habitual diet excluding wine, grapes, dark chocolate (<70%) and berries (due to their high content of resveratrol, also present in peanuts), as well as nuts (due to a similar lipid content).

### Outcome Measurements

At baseline and the end of the intervention, participants attended the research center under fasting conditions (between 8:00 and 10:30 a.m.) to have anthropometric measurements taken by trained staff. BMI was calculated as weight divided by height squared (kg/m^2^). Height was measured in the standing position using a portable stadiometer. Weight and body fat were measured using a tetrapolar OMRON BF511 bioelectrical device, with the participants wearing light clothes and no shoes. Waist circumference was measured using an inelastic flexible tape positioned equidistantly between the lowest rib and the iliac crest. Blood pressure was measured in triplicate in the sitting position using a digital monitor OMRON M6. Biochemical markers in serum and plasma (glucose and lipid profile, respectively) were measured in an external laboratory (Cerba internacional, Barcelona, Spain) using enzymatic methods. For that, blood was extracted via venipuncture into tubes containing ethylenediaminetetraacetic acid (EDTA). Serum and plasma were separated after centrifugation at 3,000 g for 10 min at 4°C and at 1,500 g for 15 min at 4°C, respectively.

In addition, diet and physical activity were recorded by trained staff through a 151-item semi-quantitative food frequency questionnaire (FFQ) and a Spanish validated version of the Minnesota Leisure-Time Physical Activity Questionnaire, respectively (12, 13). Both questionnaires were conducted at baseline and at the end of the intervention.

### Statistical Analysis

The sample size was calculated to ensure a significance level of 0.05 and statistical power of 80%, as well as 5% of loss for follow-up were included. The normality of distribution was analyzed by the Shapiro–Wilk test, and due to the Non-normality of most variables, Non-parametric tests were used. The Kruskal Wallis test followed by Dunn's *post hoc* test were applied to detect any differences between interventions at baseline. Chi-square was used for categorical variables to detect differences in participant characteristics between the three groups at baseline. A generalized estimating equation based on a Poisson regression model for repeated measures and adjusted for age and sex was used to estimate the effect of the interventions. The Wilcoxon signed-rank test was applied to evaluate any differences at the end of the study with respect to the baseline in each arm group. Continuous variables were expressed as mean ± standard deviation and categorical variables as number (*n*) and proportion (%). Differences were considered significant when the *p* value was lower than 0.05. All statistical analyses were conducted by intention-to-treat using STATA software version 16.0 (StataCorp, College Station, TX, USA).

### Meta-Analysis

#### Protocol Register

The protocol of this systematic review and meta-analysis was registered in the platform OSF (https://osf.io/jx34y/). In addition, this study was carried out according to PRISMA (Preferred Reporting Items for Systematic Reviews and Meta-Analysis, Supplementary Table 1), following the Cochrane Group recommendations (14).

#### Systematic Search and Selection of Studies

PubMed, Web of Science, Cochrane Library and Scopus databases were used for the systematic search (all years up to July 2021). Both Medical Subject Heading (MeSH) and free-text search terms were used according to the Cochrane Group recommendations. The search strategy included: (peanut OR Arachis OR Groundnut OR “Ground Nut” OR “peanut butter”) AND (“Insulin Resistance” OR “Insulin Sensitivity” OR Insulin OR Glucose OR “Glucose Intolerance” OR “Glucose Tolerance” OR “blood glucose” OR “glycemic index” OR “Waist Circumference” OR “Sagittal Abdominal Diameter” OR “Quetelet index” OR “Body Mass Index” OR adiposity OR obesity OR overweight OR “body weight” OR “weight gain” OR “weight loss” OR “body fat” OR “body composition” OR “body constitution” OR cholesterol OR Triacylglycerol OR Triglycerides OR “plasma lipid” OR “Blood Pressure” OR “Arterial Pressure” OR “Diastolic Pressure” OR “Systolic Pressure”). In addition, there were no language restrictions in the search.

The titles and abstracts identified in the systematic search were independently reviewed by I.P-M and S.H-B. Potentially relevant full texts were selected by the same two authors (I.P-M and S.H-B).

#### Selection Criteria

The inclusion criteria were the following: 1) healthy or suffering metabolic syndrome (MetS) or at high risk of MetS subjects; 2) intervention based on intake of peanuts (including high-oleic peanuts) or peanut butter (studies evaluating the effects of peanut oil consumption were excluded); 3) health outcomes that referred to anthropometric measurements, biochemical analyses (related to glucose and lipid metabolism) and clinical parameters (blood pressure); 4) randomized controlled trial (RCT) design. Details about PICOS strategy are described in Supplementary Table 2.

#### Data Extraction

After the study selection, I.P-M and S.H-B extracted the data. For each study, the following data were collected: i) author and year, ii) number and characteristics of participants, iii) study design (including intervention length), iv) control group, v) intervention group(s), vi) health outcomes [body weight, BMI, waist circumference, body fat, glucose, insulin, total cholesterol, HDL-cholesterol, LDL-cholesterol, triglycerides, total cholesterol/HDL-cholesterol and LDL-cholesterol/HDL-cholesterol, systolic blood pressure (SBP) and diastolic blood pressure (DBP)].

#### Quality Assessment

I.P-M and S.H-B independently checked the quality of the included studies. The revised Cochrane risk-of-bias tool for randomized trials (RoB-2) was used to evaluate the risk of bias in each study (15). According to the design of the RCT, the specific template of the Rob-2 was assayed: i) individually randomized parallel-group trial, ii) cluster-randomized parallel-group trial or iii) individually randomized cross-over or other matched design. The tool assesses five domains of bias: the randomization process, deviation from the intended interventions, missing outcome data, measurement of the outcome and selection of the reported result. The overall risk of bias assessment for each study was summarized within each domain. A low, unclear, or high risk of bias was established for each study considering all the domains.

In addition, I.P-M and S.H-B independently checked the quality of evidence for each outcome. Thus, the Grading of Recommendations, Assessment, Development and Evaluations (GRADE) framework (16) was assayed using the software GRADEpro. The following domains were evaluated: risk of bias, inconsistency, indirectness, imprecision, and publication bias. The overall certainty of evidence was calculated considering all the domains. Very low, low, moderate, or high certainty was established for each outcome.

### Statistical Analysis

The mean difference was calculated for each outcome considering the mean and standard deviation of control and intervention groups in the studies. For that, the data for each variable were converted to specify units. A mixture of change-from-baseline and final values were included (17). Each intervention phase of a crossover study was treated as an independent arm of a parallel study. In studies on the consumption of peanut products (peanuts, peanut butter, or high-oleic peanuts) with two or more experimental arms, a combined arm was calculated for a comparison with the control group. The meta-analysis was performed by pooling mean differences if ≥2 studies reported data for specific outcomes. Moreover, subgroup analyses were carried out according to the health status of the participants and the interventions. First, healthy subjects and patients at risk of metabolic syndrome were analyzed separately. Second, high-oleic peanuts were analyzed independently of peanut and peanut butter interventions. The random-effect model was used in all cases due to the high variability of the studies and the low number of studies meta-analyzed. The I^2^ test, Tau^2^, and 95% prediction intervals were used to evaluate the heterogeneity across studies. Finally, we estimated the dose-response effect of peanut consumption using the doresmeta package in R version 4.1.1. Meta-analyses were performed with the software Review Manager 5.4.

## Results

### Enrollment and Baseline Characteristics of Participants

Of the 90 healthy subjects that were randomized and enrolled, 63 completed the study (Supplementary Figure 1). The average age of the 63 subjects was 22.71 ± 3.13 years; around 70% were female and 36% had graduated from a 4-year degree course. At baseline, no significant differences were reported in the participant characteristics, except in HDL-cholesterol and LDL-cholesterol/HDL-cholesterol (*p* = 0.006 and *p* = 0.031, respectively) (Table 1).

**Table 1:** Participant characteristics at baseline.

	**CB (*n* = 19)**	**SRP (*n* = 21)**	**PB (*n* = 23)**	***p-value***
Female, *n* (%)	12 (63)	14 (66)	18 (78)	0.528
Age (years)	22.42 ± 3.29	22.28 ± 3.20	23.43 ± 2.90	0.247
**Education level**, ***n*** **(%)**				0.512
University students	12 (63%)	11 (52%)	11 (48%)	–
Graduated	7 (37%)	10 (48%)	12 (52%)	–
Physical activity (mets/week)	4,607 ± 1,728	4,850 ± 2,124	4,703 ± 2,381	0.954
**Anthropometric measurements**				
Body weight (kg)	63.78 ± 10.04	63.26 ± 10.12	60.10 ± 7.72	0.412
BMI (kg/m^2^)	22.59 ± 2.67	22.12 ± 3.52	22.19 ± 2.60	0.679
Waist circumference (cm)	74.68 ± 5.99	72.73 ± 8.31	71.28 ± 5.53	0.228
Body fat (%)	26.22 ± 7.99	26.66 ± 8.07	28.45 ± 7.88	0.628
**Glucose metabolism**				
Glucose (mmol/L)	4.47 ± 0.24	4.54 ± 0.44	4.59 ± 0.35	0.581
**Lipid profile**				
TG (mmol/L)	0.80 ± 0.25	0.71 ± 0.20	0.85 ± 0.35	0.341
TC (mmol/L)	4.09 ± 0.64	4.33 ± 0.52	4.60 ± 0.88	0.137
LDL-c (mmol/L)	2.30 ± 0.50	2.22 ± 0.39	2.60 ± 0.69	0.142
HDL-c (mmol/L)	1.50 ± 0.30^a^	1.75 ± 0.30^b^	1.69 ± 0.40^b^	**0.006**
TC/HDL-c	2.76 ± 0.38	2.52 ± 0.32	2.79 ± 0.57	0.056
LDL-c/HDL-c	1.56 ± 0.35^a^	1.29 ± 0.29^b^	1.59 ± 0.53^ab^	**0.031**
**Blood pressure**				
SBP (mmHg)	110 ± 11.83	111 ± 7.34	109 ± 8.87	0.451
DBP (mmHg)	70 ± 8.73	72 ± 7.63	72 ± 6.20	0.415
**Dietary intake**				
Energy (kcal/day)	2,596 ± 477.97	2,770 ± 594.50	2,705 ± 602.17	0.588
Carbohydrates (g/day)	246.74 ± 59.49	257.43 ± 80.73	241.26 ± 73.92	0.867
Sugar (g/day)	113.89 ± 41.02	115.86 ± 34.83	111.65 ± 35.04	0.906
Fiber (g/day)	38.93 ± 15.07	45.17 ± 21.95	42.12 ± 14.65	0.768
Protein (g/day)	107.75 ± 27.51	103.72 ± 29.47	110.17 ± 31.86	0.598
Total fat (g/day)	129.53 ± 28.96	144.55 ± 29.17	141.83± 35.35	0.249
SFAs (g/day)	36.81 ± 13.02	37.61 ± 10.00	38.18 ± 11.04	0.871
MUFAs (g/day)	59.46 ± 15.87	70.37 ± 16.12	69.06 ± 17.17	0.093
PUFAs (g/day)	23.59 ± 6.59	25.91 ± 6.76	23.99 ± 7.25	0.541

*Data are expressed as mean ± SD*.

*CB, control butter; SRP, skin roasted peanuts; PB, peanut butter; BMI, Body mass index; DBP, Diastolic blood pressure; MUFAs, Monounsaturated fatty acids; PUFAs, Polyunsaturated fatty acids; SFAs, Saturated fatty acids; SBP, Systolic blood pressure; TC, Total cholesterol; TG, Triglyceride; LDL-c, low-density lipoprotein cholesterol; HDL-c, high-density lipoprotein cholesterol*.

*p column refers to differences between groups at baseline. P values < 0.05 are statistically significant (a and b superscripts) and were calculated by the Kruskal–Wallis test. Values shown in bold are statistically significant*.

### Health Outcomes

Lower total cholesterol/HDL-cholesterol and LDL-cholesterol/HDL-cholesterol ratios were observed in the SRP than in the CB group (*p* = 0.019 and 0.008, respectively). A significant decrease in physical activity was reported after the CB and SRP interventions compared to baseline (*p* = 0.034 and 0.012) due to the pandemic situation, but no changes between groups were observed. No differences were observed in other lipid parameters, body composition, glucose or blood pressure. The nutritional intake had not changed after the intervention or between groups (Table 2).

**Table 2:** Health outcomes, physical activity, and nutritional intake of healthy adults from the ARISTOTLE study.

	**CB**			**SRP**			**PB**		***p^**1**^***	***p^**2**^*** ***SRP vs. CB***	***p^**2**^*** ***PB vs. CB***
	**Pre-** **intervention**	**Post-intervention**	***P^**1**^***	**Pre-** **intervention**	**Post-intervention**	***p^**1**^***	**Pre-** **intervention**	**Post-intervention**			
**Anthropometric measurements**											
Body weight (kg)	63.78 ± 10.04	63.67 ± 10.97	0.930	63.26 ± 10.12	63.13 ± 10.91	0.850	60.10 ± 7.72	59.37 ± 7.90	0.742	0.896	0.600
BMI (kg/m^2^)	22.59 ± 2.67	22.5 ± 2.93	0.895	22.12 ± 3.52	21.99 ± 3.46	0.940	22.19 ± 2.56	21.94 ± 2.71	0.835	0.982	0.672
Waist circumference (cm)	74.68 ± 5.98	73.84 ± 6.84	0.599	72.73 ± 8.31	71.81 ± 7.79	0.706	71.28 ± 5.53	70.24 ± 5.70	0.560	0.962	0.893
Body fat (%)	26.22 ± 7.99	25.66 ± 8.26	0.855	26.66 ± 8.07	26.16 ± 8.22	0.910	28.45 ± 7.88	27.77 ± 8.57	0.838	0.844	0.512
**Glucose metabolism**											
Glucose (mmol/L)	4.47 ± 0.24	4.58 ± 0.26	0.238	4.54 ± 0.43	4.76 ± 0.30	0.087	4.59 ± 0.35	4.65 ± 0.29	0.875	0.266	0.266
**Lipid profile**											
TG (mmol/L)	0.80 ± 0.25	0.79 ± 0.24	0.594	0.71 ± 0.20	0.76 ± 0.22	0.876	0.85 ± 0.35	0.81 ± 0.30	0.505	0.557	0.847
TC (mmol/L)	4.09 ± 0.64	4.23 ± 0.64	0.807	4.33 ± 0.52	4.49 ± 0.70	0.498	4.60 ± 0.88	4.66 ± 0.86	0.975	0.968	0.709
LDL-c (mmol/L)	2.30 ± 0.50	2.49 ± 0.50	0.404	2.22 ± 0.39	2.45 ± 0.44	0.150	2.60 ± 0.69	2.80 ± 0.76	0.672	0.837	0.917
HDL-c (mmol/L)	1.50 ± 0.30	1.42 ± 0.20	0.629	1.75 ± 0.30	1.68 ± 0.31	0.519	1.69 ± 0.40	1.59 ± 0.31	0.740	0.919	0.886
TC/HDL-c	2.76 ± 0.38	2.99 ± 0.40	0.121	2.52 ± 0.32	2.69 ± 0.30	0.099	2.79 ± 0.57	2.97 ± 0.62	0.207	**0.019**	0.819
LDL-c/HDL-c	1.56 ± 0.35	1.76 ± 0.37	0.125	1.29 ± 0.29	1.48 ± 0.29	0.072	1.59 ± 0.53	1.80 ± 0.61	0.191	**0.008**	0.727
**Blood pressure**											
SBP (mmHg)	110 ± 11.83	110 ± 15.65	0.715	111 ± 7.34	111 ± 18.45	0.624	109 ± 8.87	106 ± 15.00	0.317	0.982	0.982
DBP (mmHg)	70 ± 8.73	70 ± 12.83	0.693	72 ± 7.63	73 ± 12.71	0.734	72 ± 6.20	73 ± 9.38	0.886	0.487	0.487
**Physical activity**											
Physical activity (mets/week)	4,607 ± 1,728	3,330 ± 1,983	**0.034**	4,850 ± 2,124	3,269 ± 1,613	**0.012**	4,703 ± 2,381	3,736 ± 1,837	0.144	0.416	0.290
**Nutritional intake**											
Energy (kcal/day)	2,596 ± 477	2,640 ± 324	0.474	2,770 ± 594	2,663 ± 499	0.753	2,705 ± 602	2,668 ± 478	0.750	0.120	0.450
Carbohydrates (g/day)	246 ± 59.49	227 ± 46.34	0.373	257 ± 80.73	238 ± 65.18	0.443	241 ± 73.92	226 ± 53.41	0.462	0.864	0.678
Sugar	113 ± 41.02	93.25 ± 28.47	0.118	115 ± 34.83	101 ± 33.12	0.163	111 ± 35.04	95.69 ± 28.03	0.127	0.370	0.426
Fiber	38.93 ± 15.07	34.97 ± 10.55	0.457	45.17 ± 21.95	43.80 ± 18.22	0.734	42.12 ± 14.65	40.56 ± 10.07	0.818	0.202	0.302
Protein (g/day)	107 ± 27.51	115 ± 25.65	0.194	103 ± 29.47	105 ± 26.77	0.753	110 ± 31.86	111 ± 24.13	0.974	0.159	0.158
Total fat (g/day)	129 ± 28.96	148 ± 22.71	0.084	144 ± 29.17	146 ± 28.43	0.642	141 ± 35.35	151 ± 31.07	0.386	0.080	0.168
SFAs (g/day)	36.81 ± 13.02	38.04 ± 10.03	0.965	37.61 ± 10.00	36.76 ± 10.62	0.950	38.18 ± 11.04	37.37 ± 10.71	0.575	0.285	0.301
MUFAs (g/day)	59.46 ± 15.87	67.29 ± 14.62	0.088	70.37 ± 16.12	67.76 ± 15.90	0.811	69.06 ± 17.17	69.73 ± 15.96	0.957	0.200	0.141
PUFAs (g/day)	23.59 ± 6.59	20.69 ± 4.59	0.140	25.91 ± 6.76	22.45 ± 4.80	0.076	23.99 ± 7.25	21.90 ± 4.87	0.318	0.716	0.678

*Data are expressed as mean ± SD*.

*CB, control butter; SRP, skin roasted peanuts; PB, peanut butter; BMI, Body mass index; SBP, Systolic blood pressure; DBP, Diastolic blood pressure; MUFAs, Monounsaturated fatty acids; PUFAs, Polyunsaturated fatty acids; SFAs, Saturated fatty acids; TG, Triglyceride; TC, Total cholesterol; LDL-c, low-density lipoprotein cholesterol; HDL-c, high-density lipoprotein cholesterol*.

*p1 represents the p value at the end of the intervention compared to baseline, calculated by Wilcoxon's test. p2 represents the p value between SRP and PB vs. CB at 6 months adjusted by sex and age, calculated by the generalized estimating equation (GEE). p values < 0.05 are statistically significant. Values shown in bold are statistically significant*.

### Meta-Analysis

#### Selected Studies and Their Participants

A total of 4,100 articles were identified from the databases and 3,130 articles were screened after the removal of duplicates. Finally, 10 of the 29 potentially eligible full-text articles were included in the systematic review and meta-analysis. The reasons for study exclusion are set out in Supplementary Table 3. In addition, data from the ARISTOTLE study were included in this updated meta-analysis (Supplementary Figure 2). The number of selected articles dealing with each outcome was the following: 8 for body weight, 7 for BMI, 5 for body fat, 7 for waist circumference, 8 for glucose, 4 for insulin, 9 for total cholesterol, 9 for HDL-cholesterol, 9 for LDL-cholesterol, 9 for triglycerides, 5 for total cholesterol/HDL-cholesterol, 7 for LDL-cholesterol/HDL-cholesterol, 3 for SBP and 3 for DBP (Supplementary Table 4).

The data about the health outcomes of peanut interventions reported by the studies included in the systematic review and meta-analysis are presented in Supplementary Table 5. A total of 643 participants (316 males and 327 females) aged between 18 and 84 years from Asia, North America, Europa, South America, and Australia took part in these studies. Their health status was variable: healthy (*n* = 110) or suffering MetS or at high risk of MetS, with overweight or obesity, diabetes mellitus type II and hypercholesterolemia (*n* = 533). Interventions included peanuts, peanut butter and high oleic peanuts in variable concentrations and duration. The administered doses ranged between 25 and 200 g/d, with follow-up periods of 2–24 weeks. Different control diets were used: a hypocaloric diet, the habitual diet excluding peanuts (of equal or lower energy than the peanut intervention) or the American Diabetes Association meal plan without peanuts or a substitute snack (grain bar, white rice bar, candy, or almonds). In addition, an isocaloric control containing peanut oil was used in the ARISTOTLE study (free of fiber and polyphenols). The analyzed outcomes were body weight, BMI, waist circumference, body fat, glucose, insulin, total cholesterol, HDL-cholesterol, LDL-cholesterol, triglycerides, total cholesterol/HDL-cholesterol and LDL-cholesterol/HDL-cholesterol, systolic blood pressure and diastolic blood pressure. Regarding the study design, 8 parallel RCTs and 3 crossover RCTs were included Table 3).

**Table 3:** Summary of studies included in the systematic review and meta-analysis evaluating the effect of peanut product intake on health outcomes.

**Author (year)**	**Number and characteristics of participants**	**Study design (length of the intervention)**	**Control group**	**Intervention group(s)**	**Health outcomes**
Alves et al. (18)	65 overweight or obese men (18–50 years)	Parallel RCT (4 weeks)	Hypocaloric diet	Hypocaloric diet including 56 g/day of unpeeled roasted peanuts (CVP or HOP)	Body weight, BMI, waist circumference, body fat (%)
Alves et al. (19)	65 overweight or obese men (18–50 years)	Parallel RCT (4 weeks)	Hypocaloric diet	Hypocaloric diet including 56 g/day of unpeeled roasted peanuts (CVP or HOP)	Glucose, insulin, TC, LDL-c, HDL-c, triglycerides, TC/HDL-c, LDL-c/HDL-c
Barbour et al. (8)	61 overweight or obese men or postmenopausal women (50–75 years)	Crossover RCT (12 weeks)	Habitual diet without peanuts or nuts	Habitual diet adding roasted unsalted HOP: 84 g/day in men and 56 g/day in women 6 days per week	Body weight, BMI, waist circumference, body fat (%), glucose, insulin, TC, LDL-c, HDL-c, triglycerides, LDL-c/HDL-c
Claesson et al. (5)	25 healthy adults (19–30 years)	Parallel RCT (2 weeks)	20 kcal/kg/day of candy	20 kcal/kg/day of roasted and salted peanuts (~200 g/day)	Body weight, BMI, waist circumference, body fat (%), glucose, insulin, TC, LDL-c, HDL-c, triglycerides, LDL-c/HDL-c
Ghadimi Nouran et al. (20)	54 hypercholesterolaemic men (25–65 years)	Crossover RCT (4 weeks)	Habitual diet	Habitual diet adding roasted and salted peanuts (20% of total energy = 60 g/day−93 g/day)	Body weight, TC, LDL-c, HDL-c, triglycerides, TC/HDL-c, LDL-c/HDL-c, SBP, DBP
Hou et al. (21)	25 adults with type 2 diabetes Mellitus (40–80 years)	Parallel RCT (12 weeks)	Low-carbohydrate diet supplemented with unsalted almonds with skin (55 g/day in men and 45 g/day in women)	Low-carbohydrate diet supplemented with unsalted peanuts with skin (60 g/day in men and 50 g/day in women)	BMI, glucose, TC, LDL-c, HDL-c, triglycerides
Johnston et al. (18)	44 overweight or obese adults (20–65 years)	Parallel RCT (16 weeks)	40 g/day of grain bar	28 g/day of peanuts	Body weight, waist circumference, body fat (%), glucose, insulin
Kris-Etherton et al. (22)	22 healthy adults (21–54 years)	Crossover RCT (24 days)	Average American diet	MUFA-rich diet based on peanuts and peanut butter	TC, LDL-c, HDL-c, triglycerides, TC/HDL-c, LDL-c/HDL-c
Wang et al. (23)	224 adults with metabolic syndrome (MetS) or at risk of MetS (20–65 years)	Parallel RCT (12 weeks)	White rice snack bar	56 g/day of roasted salted peanuts	Body weight, BMI, waist circumference, glucose, TC, LDL-c, HDL-c, triglycerides, SBP, DBP
Wien et al. (24)	60 adults with type 2 diabetes Mellitus (34–84 years)	Parallel RCT (24 weeks)	ADA meal plan without peanuts and tree nuts	ADA meal plan + 46 g/day of salted peanuts and/or peanut butter with salt and oil (without other tree nuts)	Body weight, BMI, waist circumference, glucose, TC, LDL-c, HDL-c, triglycerides, TC/HDL-c, LDL-c/HDL-c

*ADA, American Diabetes Association; BMI, body mass index; CVP, conventional peanuts; DBP, diastolic blood pressure; HDL-c, high-density lipoprotein cholesterol; HOP, high oleic peanuts; LDL-c, low-density lipoprotein cholesterol; MUFA, monounsaturated fatty acids; RCT, randomized controlled trial; SBP, systolic blood pressure; TC, total cholesterol*.

#### Anthropometric Measurements

**Figure 1:**
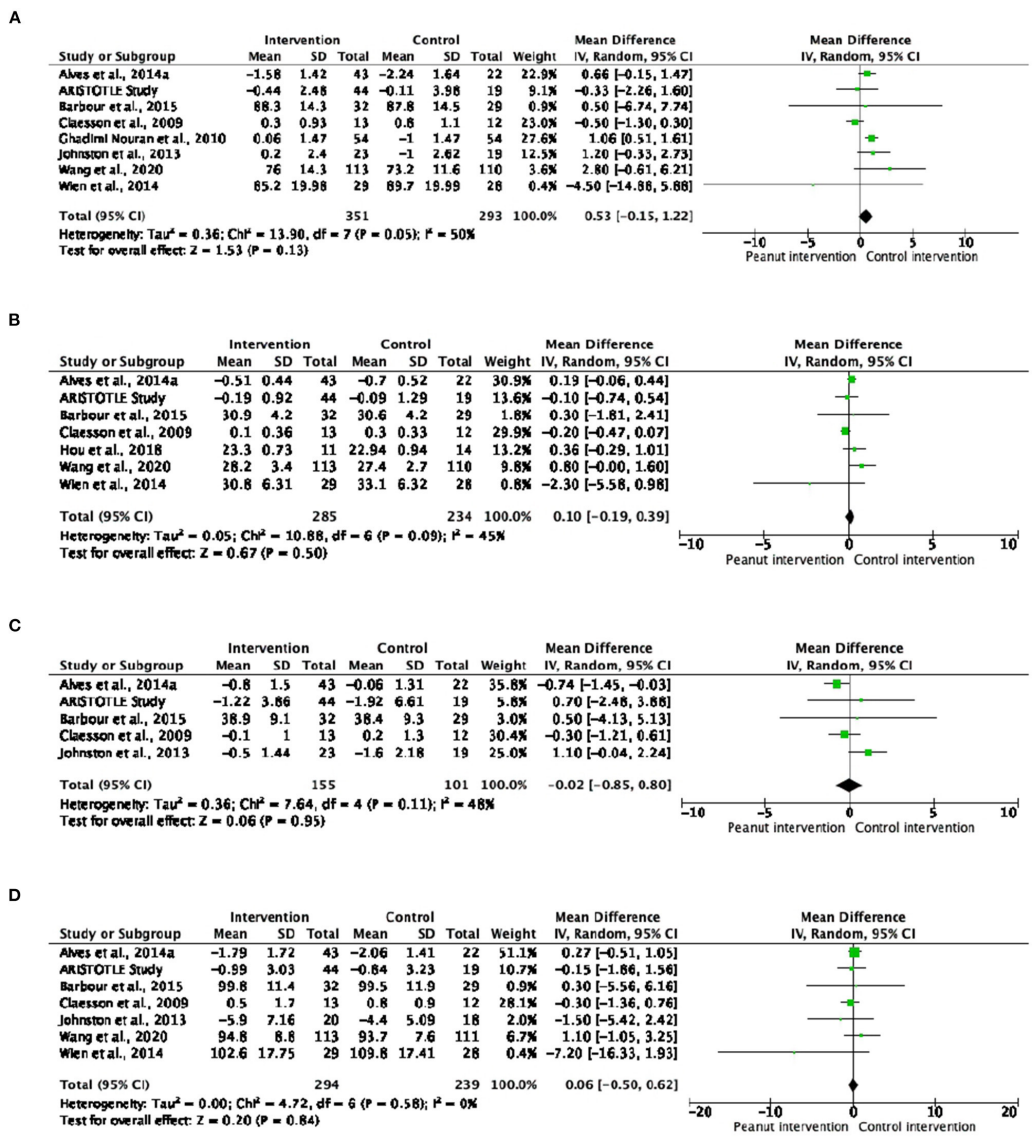
Forest plot reporting mean differences for body weight **(A)**, body mass index **(B)**, body fat **(C)** and waist circumference **(D)** associated with peanut interventions compared to control interventions.

A total of nine studies analyzed body composition parameters (body weight, BMI, body fat and/or waist circumference). In general, no significant changes were detected in the anthropometric measurements (Figure 1), but a significant increase in body weight was observed in the subjects with or at risk of MetS included in the six studies analyzed separately (MD: 0.97; 95% CI: 0.54 to 1.41; *P* < 0.0001) (Supplementary Table 6). The dose response meta-analyses showed a significant but slight effect of peanut intake (evaluated as g/day) on body weight [curve (estimate): 0.033 kg; 95% CI: 0.000 to 0.066 kg; *P* = 0.049]. No significant trends were observed for the other anthropometric parameters (Supplementary Figure 3).

#### Glucose Metabolism

**Figure 2:**
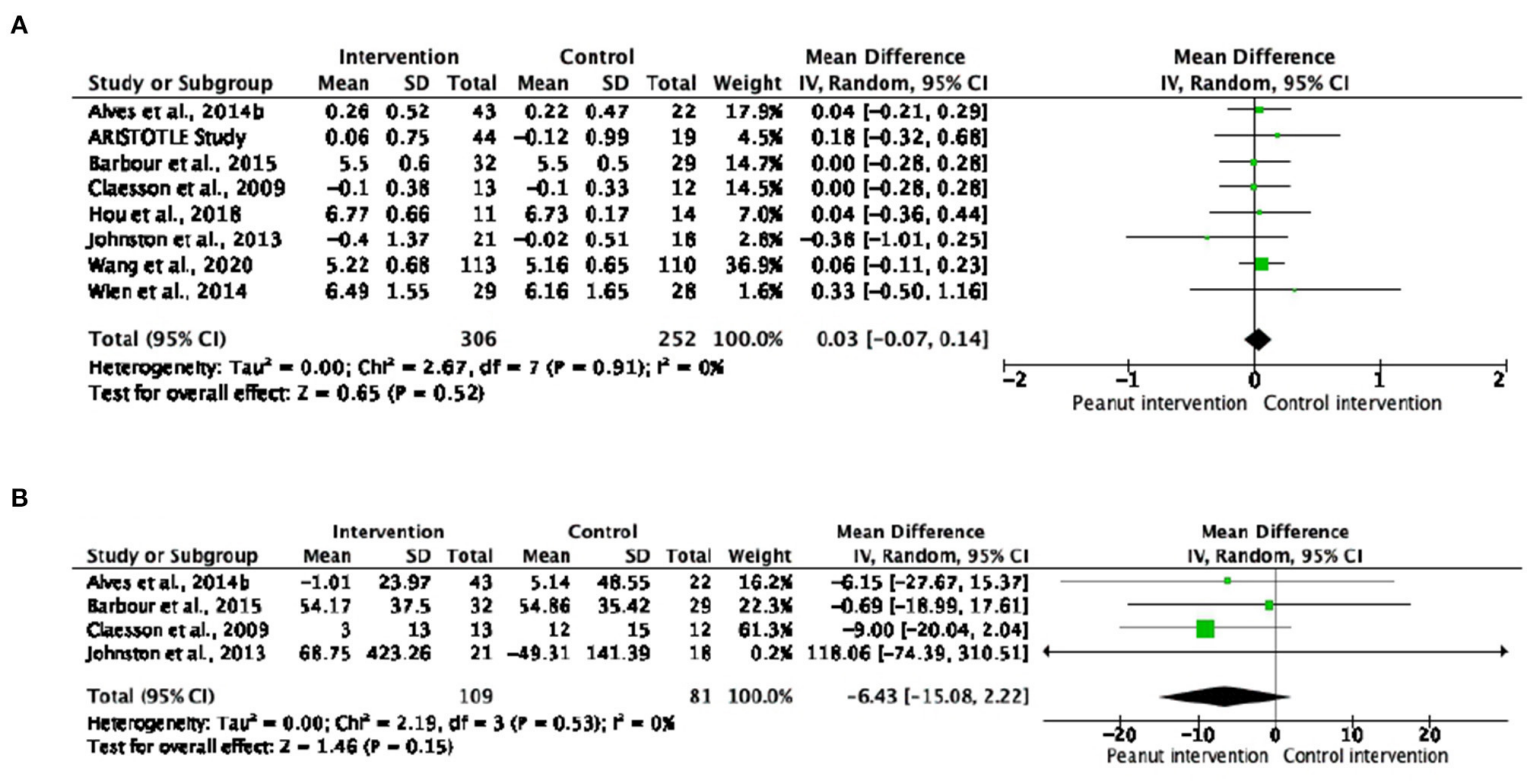
Forest plot reporting mean differences for glucose **(A)** and insulin **(B)** associated with peanut interventions compared to control interventions.

No changes were observed in fasting blood glucose or insulin in subjects that consumed peanut products compared to control interventions (Figure 2). Nor were differences found when analyzing subgroups according to health status or peanut type intake (Supplementary Tables 6, 7). Regarding the dose-response analyses, no significant effects of peanut intake on glucose metabolism were observed (Supplementary Figure 4).

#### Lipid Profile

**Figure 3:**
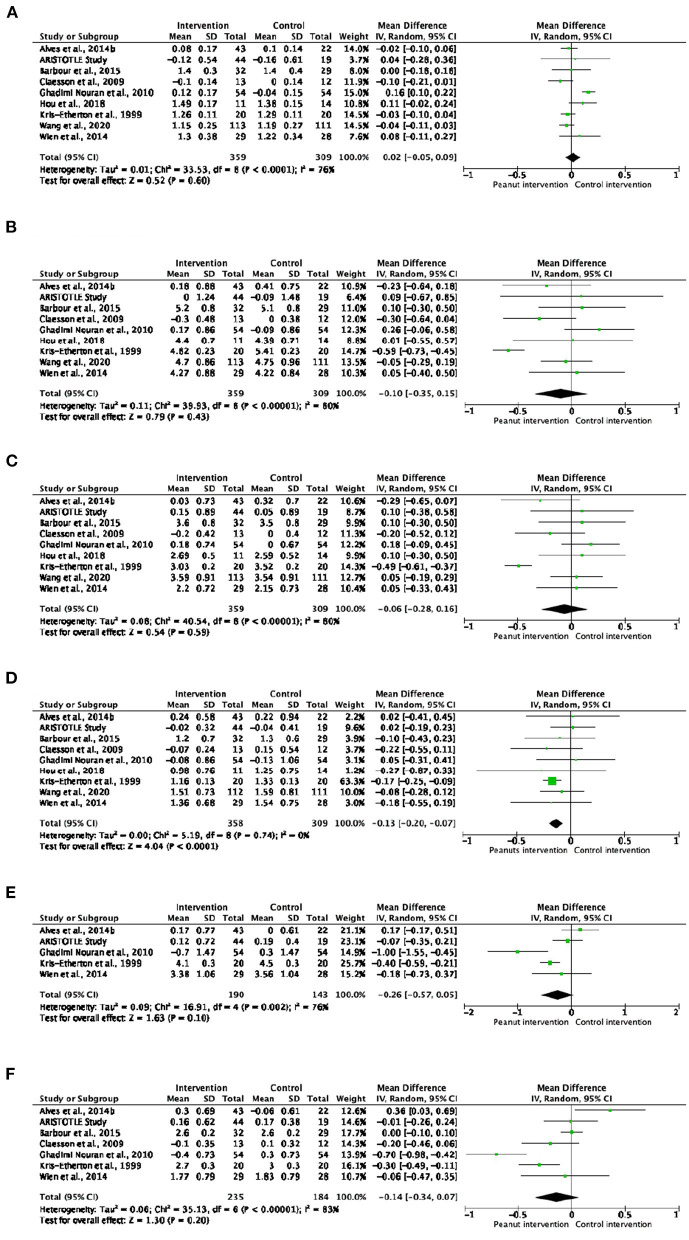
Forest plot reporting mean differences for total cholesterol **(A)**, HDL-cholesterol **(B)**, LDL-cholesterol **(C)**, triglycerides **(D)**, total cholesterol/HDL-cholesterol ratio **(E)** and LDL-cholesterol/HDL-cholesterol ratio **(F)** associated with peanut interventions compared to control interventions. HDL, high-density lipoprotein; LDL, low-density lipoprotein.

As shown in Figure 3, the level of triglycerides in blood decreased significantly after interventions with peanut products compared to the control interventions (MD: −0.13; 95% CI: −0.20 to −0.07; *p* < 0.0001). This reduction was most acute in healthy subjects (MD: −0.13; 95% CI: −0.25 to −0.00; *p* = 0.04) and in those who consumed peanuts or peanut butter (MD: −0.14; 95% CI: −0.20 to −0.07; *p* < 0.0001) (Supplementary Tables 6, 7). Although no significant changes were observed in the other lipid analytes, healthy subjects that consumed peanut products had lower total cholesterol and LDL-cholesterol/HDL-cholesterol ratio (MD: −0.40; 95% CI: −0.71 to −0.09; *p* = 0.010 and MD: −0.19; 95% CI: −0.36 to −0.01; *p* = 0.030, respectively) in comparison with control groups (Supplementary Table 6). Nevertheless, no significant trend was observed in the dose-response analyses of the effect of peanut intake on blood lipids (Supplementary Figure 5).

#### Blood Pressure

**Figure 4:**
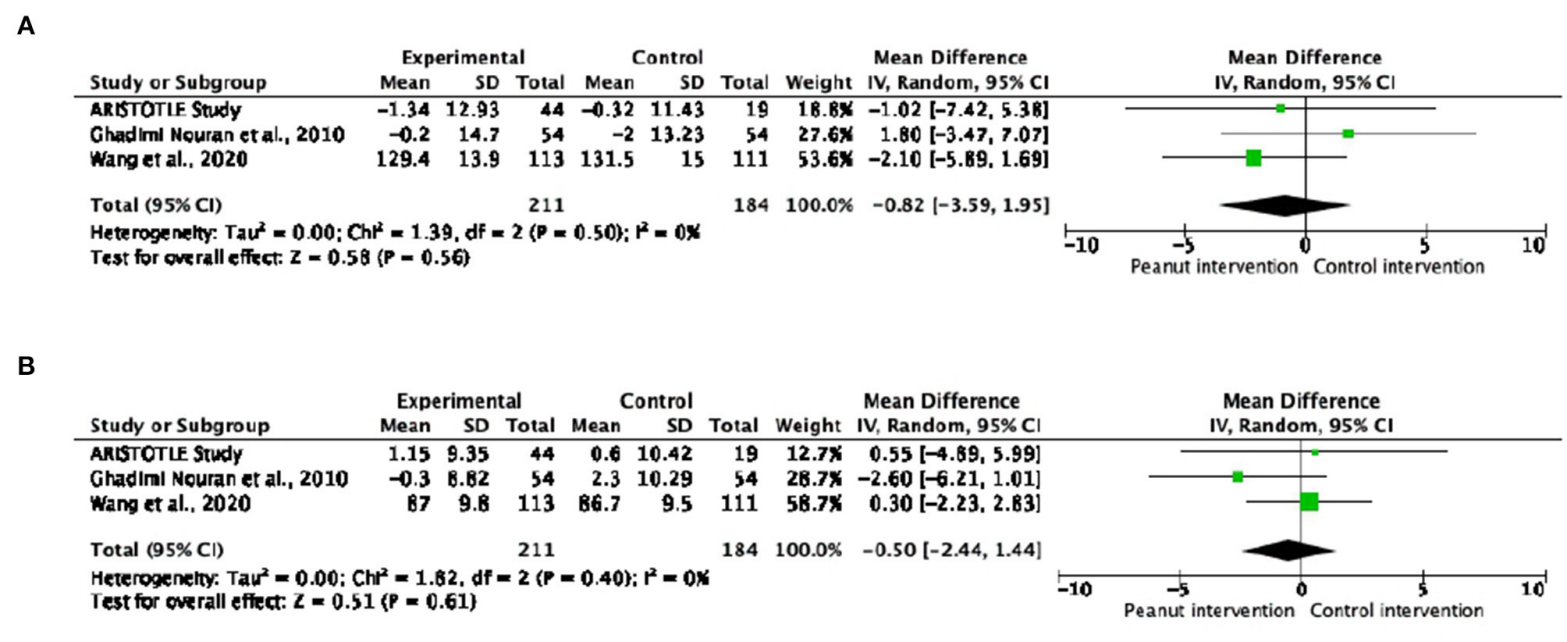
Forest plot reporting mean differences for systolic blood pressure **(A)** and diastolic blood pressure **(B)** in relation to peanut interventions compared with control interventions.

No significant changes were observed in SBP or DBP in peanut product consumers compared to the control groups (Figure 4). Similar results were obtained when analyzing subgroups according to the health status of participants and type of peanut intake (Supplementary Tables 6, 7). Regarding the dose-response analyses, no significant effects of peanut intake on blood pressure were observed (Supplementary Figure 6).

#### Quality of Studies and Overall Strength of Evidence

The overall risk of bias was high in two studies (5, 22), unclear in seven studies (8, 18, 20, 21, 24–26), as well as in the ARISTOTLE study, and low in one study (23). The main concerns regarding bias were the randomization process and outcome measurement. In addition, an unclear risk was identified in some studies regarding the deviation from the intended intervention domain (Supplementary Figure 7). The strength of evidence varied from very low to moderate, depending on the outcomes. Evidence quality for the effects of ingesting peanut products was very low regarding body fat, insulin, total cholesterol/HDL-cholesterol, DBP and SBP, and low for body weight, BMI, total cholesterol, HDL-cholesterol, LDL-cholesterol and LDL-cholesterol/HDL-cholesterol. In the case of waist circumference, glucose, and triglycerides, the quality of evidence was rated as moderate. Evidence quality was reduced by: i) heterogeneity among the participants, ii) differences in participants, comparator groups and follow-up duration, iii) small sample size (<400 participants), iv) heterogeneity in the intervention and v) bias arising from the effect estimate (Supplementary Table 8).

## Discussion

The results of the ARISTOTLE study, a randomized controlled trial, provide evidence that peanut consumption may improve lipid profiles, as the total cholesterol/HDL-cholesterol and LDL-cholesterol/HDL-cholesterol ratios were slightly lower in the SRP group compared to the CB group after the 6-month intervention. An improvement in blood lipids was also found in the meta-analysis of nine studies evaluating this cardiovascular risk factor in peanut consumers. The main finding was a reduction in triglyceride levels after peanut product consumption, this effect being greater in healthy subjects than in patients with or at high risk of MetS. The LDL-cholesterol/HDL-cholesterol ratio was also lower in healthy peanut product consumers. In addition, subgroup analyses showed that triglyceride levels were significantly lower after the interventions with peanuts and peanut butter but not high-oleic peanuts.

In agreement with our findings, Alper et al. reported a 24% lower triglyceride level in 15 normolipidemic adults after regular peanut consumption in a 30-week trial (27). Elsewhere, acute peanut intake (85 g) within a high-fat meal improved the postprandial triglyceride response and preserved endothelial function in 15 healthy overweight or obese men (28). Healthy consumers of peanuts had 7.2 and 20% less total cholesterol and triglycerides, respectively, after an 8-week intervention (3). Moreover, a recent systematic review and meta-analysis found an enhancement of HDL-cholesterol in healthy subjects consuming peanut products, particularly high-oleic peanuts, for periods longer than 12 weeks (9). In a parallel study with 118 adults who randomly consumed 56 g of peanuts in different forms, an increase in HDL-cholesterol and a reduction of the triglycerides/HDL-cholesterol ratio (considered a predictive marker for higher small dense (sd)-LDL-cholesterol and an independent predictor of cardiovascular risk) were reported Post-intervention compared to baseline (7, 29, 30). Notably, the participants with high total cholesterol, mainly those who had a high LDL-cholesterol level, experienced a significantly greater reduction in total cholesterol and LDL-cholesterol than those with normal cholesterol values. Similarly, subjects with a high triglyceride level underwent a more pronounced decrease in triglycerides (7). Consuming peanuts two or more times/week was associated with a 13% lower risk of total cardiovascular and coronary heart diseases in two large prospective cohorts of women from the Nurses' Health Study and men from the Health Professionals Follow-Up Study, but no significant associations were observed in those who consumed higher amounts of peanut butter (6). The lack of beneficial effects on the total cholesterol, LDL-cholesterol and HDL-cholesterol after peanut consumption observed in the ARISTOTLE study, highlights the importance of the analysis of atherogenic lipoproteins, and particularly sd-LDL-cholesterol, beyond lipid levels. Indeed, previous studies mention that sd-LDL-cholesterol are associated to cardiovascular diseases and closely linked to atherosclerosis formation and progression independently of LDL-cholesterol concentrations (31, 32). Therefore, to assure that peanut consumption may therefore have a positive impact on cardiovascular risk, beyond plasma lipid levels, sd-LDL-cholesterol levels must be addressed by future prospective studies.

More than half of the total lipid content in peanuts is composed of oleic acid, which is linked to better cardiovascular health (33, 34). In addition, peanuts contain specific very-long-chain saturated fatty acids (arachidic, behenic and lignoceric acids) that have been inversely associated with the risk of cardiovascular diseases and diabetes (35, 36) and we have previously found that participants from the ARISTOTLE study significantly increased the levels of these fatty acids in plasma after 6 months consuming peanut products (37, 38). Moreover, peanuts are also a good source of bioactive compounds known to be protective against cardiovascular diseases, including magnesium, folate and phytochemicals such as polyphenols and phytosterols (2).

No changes in body composition (body weight, BMI, body fat and/or waist circumference) were observed in healthy subjects in the ARISTOTLE study or meta-analysis. A slight but significant increase in body weight has been described in individuals at cardiometabolic risk. A slight increase on body weight was observed in those consuming higher amounts of peanut products, although studies report contradictory results for this effect. Similar to our findings, in a crossover randomized controlled trial, a higher body weight was observed in 54 hypercholesterolemic men consuming 60–93 g/day of peanuts for 4 weeks (20). Conversely, Alves et al. found that body fat decreased in overweight and obese subjects who consumed 56 g/day of conventional or high-oleic peanuts for 4 weeks compared to those who followed a hypocaloric diet (25). In a prospective cohort of women from the Nurses' Health Study, a marginally significant mean weight loss of 0.37 kg was found during 8 years of follow-up in those who consumed peanuts more frequently, but this trend was not associated with peanut butter intake. Similar weight loss was observed in normal weight, overweight and obese subjects (39). McKiernan et al. reported similar effects of peanut consumption on body weight independently of whether they were processed or not (7).

The incomplete absorption of fat from peanuts, which leads to less available energy, may be one of the elements protecting consumers against weight gain and body composition changes (40). Traoret et al. found that the intake of whole peanuts was associated with a higher excretion of fecal fat and energy compared to peanut butter, oil or flour (41). This loss, consistently reported by many studies, is attributed to inefficient mastication coupled with the resistance of peanut cell walls, which act as a physical barrier against the action of lipase and limit the bioaccessibility of peanut lipids and energy (42). Several authors describe a greater sensation of fullness and satiety after peanut intake (19, 43). A study even observed that peanuts consumed as a snack had a greater compensatory effect on energy intake than when consumed with a meal (44). In addition, regularly consumed peanut products could be replacing sugary or processed snacks (45).

Regarding glucose metabolism, no significant effects on glucose or insulin were observed in this research, in accordance with previous studies (5, 9, 28, 46, 47). However, the consumption of peanut butter five times or more per week reduced the incidence of diabetes by 21% in a prospective cohort of women from the Nurses' Health Study (48). The addition of 32 g of peanut butter to a high-glycemic index meal reduced the fasting blood glucose and overall glycemic response in 16 healthy adults (49). Reis et al. also showed a reduced glycemic response, depending on the processing and form of the consumed peanuts (4, 50). The fat in peanut products could delay gastric emptying and reduce the rate of glucose uptake into the circulation and the insulin response (51). Moreover, due to their high concentration of fiber, peanuts may be considered as prebiotics, which can reduce the glycemic index and glycemic load (52).

The three selected studies evaluating blood pressure after a peanut intervention did not report any differences from the control group. Supporting these findings, other studies indicate that peanut consumption has no significant effect on SBP or DBP (53). In contrast, a randomized clinical trial observed that daily peanut consumption significantly reduced DBP, but did not alter SBP (47). Peanuts are a rich source of polyphenol compounds that can affect blood pressure (54). A study administering peanut sprout extract, which has a higher resveratrol content than peanuts, observed a significant reduction in SBP (55). In addition, peanuts are a rich source of protein, predominantly arginine, which is reported to improve endothelial function through nitric oxide release (56).

The ARISTOTLE study has several strengths, including its randomized and controlled design and its focus on the impact of peanut and peanut butter intake on the health of young healthy adults. Moreover, the peanut butter used in the study consisted exclusively of peanuts and sea salt, unlike other peanut butters that contain saturated fats as added ingredients. The main strong point of the systematic review and meta-analysis is their concentration on randomized controlled trials, including a new clinical trial, that have studied the effect of peanut consumption on metabolic syndrome.

The limitations of this research include the relatively small sample size of the ARISTOTLE study (19 to 23 individuals in each intervention) and although the sample size was calculated to assure 80% of statistical power, this value decreased to 70% due to dropouts and secondary outcomes analyzed in this manuscript. Also, the control group was based on peanut oil, as the hypothesis of the study was that the health benefits of peanuts are due to prebiotic substances, namely, polyphenols and fiber. On the other hand, the major limitation of the systematic review and meta-analysis is the heterogeneity of participants, comparator groups and follow-up periods in the included studies, which reduces evidence quality. The evidence for our major finding, that peanut product consumption improves the lipid profile, was rated as moderately strong in the case of triglycerides. However, the evidence for the impact of peanut consumption on the other outcomes (body composition, blood lipids, glucose metabolism and blood pressure) ranged between moderate and very low. Intervention effects can vary depending on the participant health status, so a strong point of the analysis is that it was also conducted on subgroups (healthy subjects vs. those at a high risk of or suffering cardiometabolic conditions). Moreover, interventions with peanuts/peanut butter and high-oleic peanuts were analyzed separately to identify possible differences. Other factors that may have influenced the results include peanut processing and/or the use of additives (i.e., salted *vs*. unsalted, roasted *vs*. raw, skinned *vs*. Non-skinned peanuts). The results may also be inconclusive due to the variability of control groups among the studies. Another potential limitation is the unclear risk of bias reported in studies, associated with the randomization process, outcome measurement and deviation from the intended intervention domain.

## Conclusions

In conclusion, this meta-analysis of randomized controlled trials, including novel results from the ARISTOTLE study, provides moderate evidence that peanut consumption has beneficial effects on triglycerides and tends to improve blood lipid values in general. However, no changes in body weight, glucose metabolism and blood pressure were observed. Although peanuts are energy-dense, their consumption does not promote weight gain in healthy subjects, and they can be incorporated into a dietary pattern to improve health. To gain more knowledge about the effects of peanut products on cardiometabolic risk factors, more carefully designed studies in larger populations are needed.

## Data Availability Statement

The raw data supporting the conclusions of this article will be made available by the authors, without undue reservation.

## Author Contributions

RL-R and SH-B designed the study. IP-M and SH-B collected data, performed statistical analysis, interpreted results, and drafted the manuscript. RL-R and MG-F interpreted the study results and performed the writing review of the manuscript. All authors contributed to the article and approved the submitted version.

## Funding

This work was supported by the funding from the Peanut Institute 2019, CICYT under Grant PID2020-114022RB-I00, AGL2016- 75329-R, CIBEROBN from the Instituto de Salud Carlos III, ISCIII from the Ministerio de Ciencia, Innovación y Universidades (AEI/FEDER, UE), and Generalitat de Catalunya (GC) under Grant 2017SGR 196. None of the funders had a role in the study design, implementation, analysis or interpretation of the data, or the writing of the manuscript.

## Conflict of Interest

RL-R reports receiving lecture fees from Cerveceros de España; and receiving lecture fees and travel support from Adventia. Nevertheless, these foundations were not involved in the study design, the collection, analysis and interpretation of data, the writing of the manuscript or the decision to submit the manuscript for publication. The remaining authors declare that the research was conducted in the absence of any commercial or financial relationships that could be construed as a potential conflict of interest.

## Publisher's Note

All claims expressed in this article are solely those of the authors and do not necessarily represent those of their affiliated organizations, or those of the publisher, the editors and the reviewers. Any product that may be evaluated in this article, or claim that may be made by its manufacturer, is not guaranteed or endorsed by the publisher.
