# Supplementary material for: Effect of Peanut Consumption on Cardiovascular Risk Factors: A Randomized Clinical Trial and Meta-Analysis
Source: Front Nutr. 2022 Apr 1;9:853378. doi: 10.3389/fnut.2022.853378 (PMC9011914; doi:10.3389/fnut.2022.853378)
Supplement: Supplementary file 1 [file Data_Sheet_1.docx]

Supplementary Material

**Supplementary Table 1.** PRISMA checklist of systematic review and meta-analysis.

| **Section and Topic** | **Item #** | **Checklist item** | **Location where item is reported** |
| --- | --- | --- | --- |
| **TITLE** | | |  |
| Title | 1 | Identify the report as a systematic review. | Page 1 |
| **ABSTRACT** | | |  |
| Abstract | 2 | See the PRISMA 2020 for Abstracts checklist. | Page 1 |
| **INTRODUCTION** | | |  |
| Rationale | 3 | Describe the rationale for the review in the context of existing knowledge. | Page 2 |
| Objectives | 4 | Provide an explicit statement of the objective(s) or question(s) the review addresses. | Page 2 |
| **METHODS** | | |  |
| Eligibility criteria | 5 | Specify the inclusion and exclusion criteria for the review and how studies were grouped for the syntheses. | Page 4 Supplementary table 1 |
| Information sources | 6 | Specify all databases, registers, websites, organisations, reference lists and other sources searched or consulted to identify studies. Specify the date when each source was last searched or consulted. | Page 3 |
| Search strategy | 7 | Present the full search strategies for all databases, registers and websites, including any filters and limits used. | Page 3-4 |
| Selection process | 8 | Specify the methods used to decide whether a study met the inclusion criteria of the review, including how many reviewers screened each record and each report retrieved, whether they worked independently, and if applicable, details of automation tools used in the process. | Page 4 |
| Data collection process | 9 | Specify the methods used to collect data from reports, including how many reviewers collected data from each report, whether they worked independently, any processes for obtaining or confirming data from study investigators, and if applicable, details of automation tools used in the process. | Page 4 |
| Data items | 10a | List and define all outcomes for which data were sought. Specify whether all results that were compatible with each outcome domain in each study were sought (e.g. for all measures, time points, analyses), and if not, the methods used to decide which results to collect. | Page 4 |
|  | 10b | List and define all other variables for which data were sought (e.g. participant and intervention characteristics, funding sources). Describe any assumptions made about any missing or unclear information. | Table 4 |
| Study risk of bias assessment | 11 | Specify the methods used to assess risk of bias in the included studies, including details of the tool(s) used, how many reviewers assessed each study and whether they worked independently, and if applicable, details of automation tools used in the process. | Page 4 |
| Effect measures | 12 | Specify for each outcome the effect measure(s) (e.g. risk ratio, mean difference) used in the synthesis or presentation of results. | Siplemmentary table 5 |
| Synthesis methods | 13a | Describe the processes used to decide which studies were eligible for each synthesis (e.g. tabulating the study intervention characteristics and comparing against the planned groups for each synthesis (item #5)). | Page 6 & Table 3 |
|  | 13b | Describe any methods required to prepare the data for presentation or synthesis, such as handling of missing summary statistics, or data conversions. | Page 4 - 5 |
|  | 13c | Describe any methods used to tabulate or visually display results of individual studies and syntheses. |  |
|  | 13d | Describe any methods used to synthesize results and provide a rationale for the choice(s). If meta-analysis was performed, describe the model(s), method(s) to identify the presence and extent of statistical heterogeneity, and software package(s) used. | Page 4 |
|  | 13e | Describe any methods used to explore possible causes of heterogeneity among study results (e.g. subgroup analysis, meta-regression). | Page 5 |
|  | 13f | Describe any sensitivity analyses conducted to assess robustness of the synthesized results. | Page 5 |
| Reporting bias assessment | 14 | Describe any methods used to assess risk of bias due to missing results in a synthesis (arising from reporting biases). | Page 4 |
| Certainty assessment | 15 | Describe any methods used to assess certainty (or confidence) in the body of evidence for an outcome. | Page 4 |
| **RESULTS** | | |  |
| Study selection | 16a | Describe the results of the search and selection process, from the number of records identified in the search to the number of studies included in the review, ideally using a flow diagram. | Page 5 & Supplementary figure 2 |
|  | 16b | Cite studies that might appear to meet the inclusion criteria, but which were excluded, and explain why they were excluded. | Page 5 & supplementary table 3 |
| Study characteristics | 17 | Cite each included study and present its characteristics. | Table 3 |
| Risk of bias in studies | 18 | Present assessments of risk of bias for each included study. | Page 7 & Supplementary figure 7 |
| Results of individual studies | 19 | For all outcomes, present, for each study: (a) summary statistics for each group (where appropriate) and (b) an effect estimate and its precision (e.g. confidence/credible interval), ideally using structured tables or plots. | Figures 1, 2, 3, 4 |
| Results of syntheses | 20a | For each synthesis, briefly summarise the characteristics and risk of bias among contributing studies. | Supplementary figure 7 |
|  | 20b | Present results of all statistical syntheses conducted. If meta-analysis was done, present for each the summary estimate and its precision (e.g. confidence/credible interval) and measures of statistical heterogeneity. If comparing groups, describe the direction of the effect. | Page 6 & Figures 1, 2, 3, 4 |
|  | 20c | Present results of all investigations of possible causes of heterogeneity among study results. | Supplementary table 8 |
|  | 20d | Present results of all sensitivity analyses conducted to assess the robustness of the synthesized results. | Page 6 & Supplementary table 6 & 7 |
| Reporting biases | 21 | Present assessments of risk of bias due to missing results (arising from reporting biases) for each synthesis assessed. | Page 7 & supplementary table 8 |
| Certainty of evidence | 22 | Present assessments of certainty (or confidence) in the body of evidence for each outcome assessed. | Page 7 & supplementary table 8 |
| **DISCUSSION** | | |  |
| Discussion | 23a | Provide a general interpretation of the results in the context of other evidence. | Page 7 - 9 |
|  | 23b | Discuss any limitations of the evidence included in the review. | Page 9 |
|  | 23c | Discuss any limitations of the review processes used. | Page 9 |
|  | 23d | Discuss implications of the results for practice, policy, and future research. | Page 9 |
| **OTHER INFORMATION** | | |  |
| Registration and protocol | 24a | Provide registration information for the review, including register name and registration number, or state that the review was not registered. | Page 3 |
|  | 24b | Indicate where the review protocol can be accessed, or state that a protocol was not prepared. | Page 3 |
|  | 24c | Describe and explain any amendments to information provided at registration or in the protocol. |  |
| Support | 25 | Describe sources of financial or non-financial support for the review, and the role of the funders or sponsors in the review. | Page 10 |
| Competing interests | 26 | Declare any competing interests of review authors. | Page 10 |
| Availability of data, code and other materials | 27 | Report which of the following are publicly available and where they can be found: template data collection forms; data extracted from included studies; data used for all analyses; analytic code; any other materials used in the review. | Page 14 |

**Supplementary Table 2.** PICOS strategy of systematic review and meta-analysis.

| P | Population | Healthy or suffering MetS or at high risk of MetS subjects |
| --- | --- | --- |
| I | Intervention | Peanut products, including peanuts, high oleic peanuts and peanut butter |
| C | Comparator | Control (e.g. habitual diet or other control interventions) |
| O | Outcomes | Cardiometabolic risk factors (body weight, BMI, waist circumference, body fat, glucose, insulin, total cholesterol, HDL-c, LDL-c, triglycerides, total cholesterol/HDL-c, LDL-c/HDL-c, SBP and DBP) |
| S | Study design | Systematic review and meta-analysis of randomized controlled trials, considering parallel and crossover designs. |

BMI: body mass index ; DBP: diastolic blood pressure; HDL-c: high-density lipoprotein cholesterol, LDL-c: low-density lipoprotein cholesterol, MetS: metabolic syndrome; SBP: systolic blood pressure.

**Supplementary Table 3.** Studies excluded from the systematic review and meta-analysis and the reasons for their exclusion.

| **Studies** | **Reason for exclusion** | | | |
| --- | --- | --- | --- | --- |
| **Reference** | **Not a randomized controlled trial** | **No relevant outcomes reported** | **No specific exposure** | **Other*** |
| Alper *et al.* 2002 (1) | x |  |  |  |
| Alper *et al.* 2003 (2) | x |  |  |  |
| Alves *et al.* 2014 (3) |  | x |  |  |
| Barbour *et al.* 2017 (4) |  | x |  |  |
| Devitt *et al.* 2011 (5) |  | x |  |  |
| Johnston *et al.* 2005 (6) | x |  |  |  |
| Jones *et al.* 2014 (7) | x |  |  |  |
| Lilly *et al.* 2019 (8) | x |  |  |  |
| Liu *et al.* 2017 (9) | x |  |  |  |
| Lokko *et al.* 2007 (10) | x |  |  |  |
| McKiernan *et al.* 2010 (11) | x |  |  |  |
| Moreira *et al.* 2016 (12) | x |  |  |  |
| O’Byrne *et al.* 1997 (13) | x |  |  |  |
| Reis *et al.* 2011 (14) |  | x |  |  |
| Reis *et al.* 2013 (15) | x |  |  |  |
| Shively *et al.* 1986 (16) | x |  |  |  |
| Caldas *et al.* 2020 (17) |  |  |  | x |
| Tan *et al.* 2018 (18) |  |  | x |  |
| Traoret *et al.* (19) |  | x |  |  |
| *Results considered unclear because they disagree with previously published results derived from the same analyses. | | | | |

**Supplementary Table 4:** Selected articles for each outcome included in the meta-analysis.

| **Studies** | **Outcome measured** | | | | | | | | | | | | | |
| --- | --- | --- | --- | --- | --- | --- | --- | --- | --- | --- | --- | --- | --- | --- |
| **Reference** | **Body weight** | **BMI** | **Body fat** | **WC** | **Glucose** | **Insulin** | **TC** | **HDL-c** | **LDL** | **TG** | **TC/HDL-c** | **LDL-c/HDL-c** | **SBP** | **DBP** |
| Alves *et al.* 2014a (20) | x | x | x | x |  |  |  |  |  |  |  |  |  |  |
| Alves *et al.*2014b (21) |  |  |  |  | x | x | x | x | x | x | x | x |  |  |
| Barbour *et al*. 2015 (22) | x | x | x | x | x | x | x | x | x | x |  | x |  |  |
| Claesson *et al.* 2009 (23) | x | x | x | x | x | x | x | x | x | x |  | x |  |  |
| Ghadimi Nouran *et al.* 2010 (24) | x |  |  |  |  |  | x | x | x | x | x | x | x | x |
| Hou *et al*. 2018 (25) |  | x |  |  | x |  | x | x | x | x |  |  |  |  |
| Johnston *et al.* 2013 (26) | x |  | x | x | x | x |  |  |  |  |  |  |  |  |
| Kris-Etherton *et al*. 1999 (27) |  |  |  |  |  |  | x | x | x | x | x | x |  |  |
| Wang *et al*. 2020 (28) | x | x |  | x | x |  | x | x | x | x |  |  | x | x |
| Wien *et al.* 2014 (29) | x | x |  | x | x |  | x | x | x | x | x | x |  |  |
| ARISTOTLE study | x | x | x | x | x |  | x | x | x | x | x | x | x | x |
| BMI: body mass index; HDL-c: high-density lipoprotein cholesterol; DBP: diastolic blood pressure; LDL-c: low-density lipoprotein cholesterol; SBP: systolic blood pressure; TC: total cholesterol; TG: triglyceride; WC: waist circumference. | | | | | | | | | | | | | | |

Supplementary Table 5. Summary of the meta-analysis results regarding the effect of total peanut intake (peanuts, peanut butter, and high-oleic peanuts) on health outcomes in all subjects.

| **Outcome** | **N#** | **HR (95%CI)** | ***I^2^ (%)*** | ***Tau^2^*** | **95% PI** |
| --- | --- | --- | --- | --- | --- |
| Body weight | 8 | 0.53 (-0.15, 1.22) | 50 | 0.36 | (-1.17, 2.23) |
| BMI | 7 | 0.10 (-0.19, 0.39) | 45 | 0.05 | (-0.59, 0.79) |
| Body fat | 5 | -0.02 (-0.85, 0.80) | 48 | 0.36 | (-2.35, 2.31) |
| WC | 7 | 0.06 (-0.50, 0.62) | 0 | 0.00 | (-2.09, 2.21) |
| Glucose | 8 | 0.03 (-0.07, 0.14) | 0 | 0.00 | (-1.07, 0.17) |
| Insulin | 4 | -6.43 (-15.08, 2.22) | 0 | 0.00 | (-25.42, 12.56) |
| TC | 9 | 0.02 (-0.05, 0.09) | 76 | 0.01 | (-0.23, 0.27) |
| HDL-c | 9 | -0.10 (-0.35, 0.15) | 80 | 0.11 | (-0.94, 0.74) |
| LDL-c | 9 | -0.06 (-0.28, 0.16) | 80 | 0.08 | (-0.78, 0.66) |
| TG | 9 | -0.13 (-0.20, -0.07) | 0 | 0.00 | (-0.20, -0.06) |
| TC/HDL-c | 5 | -0.26 (-0.57, 0.05) | 76 | 0.09 | (-1.34, 0.82) |
| LDL-c/HDL-c | 7 | -0.14 (-0.34, 0.07) | 83 | 0.06 | (-0.83, 0.55) |
| SBP | 3 | -0.82 (-3.59, 1.95) | 0 | 0.00 | (-18.78, 17.14) |
| DBP | 3 | -0.50 (-2.44, 1.44) | 0 | 0.00 | (-13.08, 12.08) |
| BMI: body mass index; HDL-c: high-density lipoprotein cholesterol; DBP: diastolic blood pressure; LDL-c: low-density lipoprotein cholesterol; SBP: systolic blood pressure; TC: total cholesterol; TG: triglyceride; WC: waist circumference. | | | | | |

**Supplementary Table 6*.*** Summary of meta-analysis results regarding the effect of total peanut intake (peanuts, peanut butter, and high-oleic peanuts) on health outcomes in healthy individuals and those with or at high risk of metabolic syndrome.

|  | **Healthy subjects** | | | | | **Subjects with or at risk of metabolic syndrome** | | | | |
| --- | --- | --- | --- | --- | --- | --- | --- | --- | --- | --- |
| **Outcome** | **N#** | **HR (95%CI)** | ***I^2^ (%)*** | ***Tau^2^*** | **95% PI** | **N#** | **HR (95%CI)** | ***I^2^ (%)*** | ***Tau^2^*** | **95% PI** |
| Body weight | 2 | -0.48  (-1.22, 0.27) | 0 | 0.00 | - | 6 | 0.97  (0.54, 1.41) | 0 | 0.00 | (0.35, 1.59) |
| BMI | 2 | -0.18  (-0.43, 0.06) | 0 | 0.00 | - | 5 | 0.29  (-0.02, 0.61) | 7 | 0.01 | (-0.32, 0.90) |
| Body fat | 2 | -0.22  (-1.10, 0.66) | 0 | 0.00 | - | 3 | 0.17  (-1.37, 1.70) | 68 | 1.08 | (-16.34, 16.68) |
| WC | 2 | -0.26  (-1.16, 0.64) | 0 | 0.00 | - | 5 | 0.25  (-0.60, 1.11) | 0 | 0.00 | (-1.15, 1.65) |
| Glucose | 2 | 0.04  (-0.20, 0.29) | 0 | 0.00 | - | 6 | 0.03  (-0.09, 0.16) | 0 | 0.00. | (-0,15, 0,21) |
| Insulin | 1 | -9  (-20.04, 2.04) | - | - | - | 3 | -1.42  (-16.93, 14.09) | 0 | 0.00 | (-101,97, 99,13) |
| TC | 3 | -0.40  (-0.71, -0.09) | 60 | 0.04 | (-3.64, 2.84) | 6 | 0.04  (-0.11, 0.18) | 0 | 0.00 | (-0.16, 0.24) |
| HDL-c | 3 | -0.05  (-0.10, 0.01) | 0 | 0.00 | (-0.44, 0.34) | 6 | 0.05  (-0.04, 0.14) | 78 | 0.01 | (-0.26, 0.36) |
| LDL-c | 3 | -0.26  (-0.59, 0.07) | 74 | 0.06 | (-4.04, 3.52) | 6 | 0.07  (-0.07, 0.20) | 0 | 0.00 | (-0.11, 0.25) |
| TG | 3 | -0.13  (-0.25, -0.00) | 33 | 0.00 | (-0.97, 0.71) | 6 | -0.08  (-0.22, 0.05) | 0 | 0.00 | (-0.26, 0.10) |
| TC/HDL-c | 2 | -0.25  (-0.57, 0.07) | 73 | 0.04 | - | 3 | -0.32  (-1.00, 0.36) | 81 | 0.29 | (-8.46, 7.82) |
| LDL-c/HDL-c | 3 | -0.19  (-0.36, -0.01) | 40 | 0.01 | (-1.92, 1.54) | 4 | -0.11  (-0.51, 0.28) | 88 | 0.14 | (-1.93, 1.71) |
| SBP | 1 | -1.02  (-7.42, 5.38) | - | - | - | 2 | -0.60  (-4.32, 3.12) | 28 | 2.12 | - |
| DBP | 1 | 0.55  (-4.89, 5.99) | - | - | - | 2 | -0.85  (-3.63, 1.93) | 40 | 1.67 | - |
| BMI: body mass index; HDL-c: high-density lipoprotein cholesterol; DBP: diastolic blood pressure; LDL-c: low-density lipoprotein cholesterol; SBP: systolic blood pressure; TC: total cholesterol; TG: triglyceride; WC: waist circumference. | | | | | | | | | | |

Supplementary Table 7. Summary of meta-analysis results regarding the effect of intake of peanuts/peanut butter and high-oleic peanuts on health outcomes in all subjects.

|  | **Peanut and peanut butter intervention** | | | | | **High-oleic peanut intervention** | | | | |  |
| --- | --- | --- | --- | --- | --- | --- | --- | --- | --- | --- | --- |
| **Outcome** | **N#** | **HR (95%CI)** | ***I^2^ (%)*** | ***Tau^2^*** | **95% PI** | **N#** | **HR (95%CI)** | ***I^2^ (%)*** | ***Tau^2^*** | **95% PI** | |
| Body weight | 7 | 0.55  (-0.22, 0.45) | 57 | 0.49 | (-1,52, 2,62) | 2 | 0.54  (-0.61, 1.69) | 0 | 0.00 | - | |
| BMI | 6 | 0.11  (-0.22, 0.45) | 53 | 0.08 | (-0,81, 1,03) | 2 | 0.15  (-0.21, 0.51) | 0 | 0.00 | - | |
| Body fat | 4 | 0.07  (-0.77, 0.90) | 44 | 0.31 | (-2.94, 3.08) | 2 | -0.17  (-0.85, 0.51) | 0 | 0.00 | - | |
| WC | 6 | -0.10  (-0.76, 0.56) | 0 | 0.00 | (-1.03, 0.83) | 2 | 0.47  (-0.58, 1.52) | 0 | 0.00 | - | |
| Glucose | 7 | 0.04  (-0.17, 0.25) | 0 | 0.00 | (-0.24, 0.32) | 2 | 0.04  (-0.17, 0.25) | 0 | 0.00. | - | |
| Insulin | 3 | -7.78  (-18.14, 2.58) | 0 | 0.00 | (-74.94, 59.38) | 2 | -3.35  (-19.05, 12.36) | 0 | 0.00 | - | |
| TC | 8 | -0.13  (-0.41, 0.14) | 81 | 0.11 | (-1.01, 0.75) | 2 | 0.03  (-0.31, 0.36) | 0 | 0.00 | - | |
| HDL-c | 8 | 0.02  (-0.06, 0.10) | 79 | 0.01 | (-0.24, 0.28) | 2 | -0.02  (-0.10, 0.07) | 0 | 0.00 | - | |
| LDL-c | 8 | -0.07  (-0.31, 0.17) | 82 | 0.09 | (-0.86, 0.72) | 2 | 0.04  (-0.41, 0.32) | 18 | 0.01 | - | |
| TG | 8 | -0.14  (-0.20, -0.07) | 0 | 0.00 | (-0.23, -0.05) | 2 | -0.07  (-0.36, 0.22) | 0 | 0.00 | - | |
| TC/HDL-c | 5 | -0.26  (-0.58, 0.07) | 76 | 0.09 | (-1.35, 0.83) | 1 | 0.07  (-0.46, 0.60) | - | - | - | |
| LDL-c/HDL-c | 6 | -0.18  (-0.43, 0.06) | 77 | 0.07 | (-0.99, 0.63) | 2 | 0.11  (-0.22, 0.44) | 55 | 0.04 | - | |
| SBP | 3 | -0.82  (-3.59, 1.95) | 0 | 0.00 | (-18.78, 17.14) | - | - | - | - | - | |
| DBP | 3 | -0.50  (-2.44, 1.44) | 0 | 0.00 | (-13.08, 12.08) | - | - | - | - | - | |
| BMI: body mass index; HDL-c: high-density lipoprotein cholesterol; DBP: diastolic blood pressure; LDL-c: low-density lipoprotein cholesterol; SBP: systolic blood pressure; TC: total cholesterol; TG: triglyceride; WC: waist circumference. | | | | | | | | | | |  |

**Supplementary Table 8.** Certainty of each outcome according to the GRADE tool

| **Outcome** | **Anticipated absolute effects ^*^ (95% CI)** | | **Relative effect**  **(95% CI)** | **№ of participants**  **(studies)** | **Certainty**  **(GRADE)** |
| --- | --- | --- | --- | --- | --- |
|  | **Risk with control (no peanuts)** | **Risk with Peanuts** |  |  |  |
| **Body weight (kg)**  Follow-up: 2-24 weeks | The mean body weight was **79.3** kg | MD **0.53 higher**  (0.15 lower to 1.22 higher) |  | 644  (8 RCTs) | ⊕⊕⊝⊝  LOW ^1,2^ |
| **BMI (kg/m^2^)**  Follow-up: 2-24 weeks | The mean BMI was **26.9** kg/m^2^ | MD **0.1 higher**  (0.19 lower to 0.39 higher) |  | 519  (7 RCTs) | ⊕⊕⊝⊝  LOW ^1,2^ |
| **Waist circumference (cm)**  Follow-up: 2-24 weeks | The mean waist circumference was **92.5** cm | MD **0.06 higher**  (0.5 lower to 0.62 higher) |  | 533  (7 RCTs) | ⊕⊕⊕⊝  MODERATE ^2^ |
| **Body fat (%)**  Follow-up: 2-24 weeks | The mean body fat was **32.7**% | MD **0.02 lower**  (0.85 lower to 0.8 higher) |  | 256  (5 RCTs) | ⊕⊝⊝⊝  VERY LOW ^2,3,4,5^ |
| **Glucose (mmol/L)**  Follow-up: 2-24 weeks | The mean glucose was **5.4** mmol/L | MD **0.03 higher**  (0.07 lower to 0.14 higher) |  | 558  (8 RCTs) | ⊕⊕⊕⊝  MODERATE ^2^ |
| **Insulin (pmol/L)**  Follow-up: 2-12 weeks | The mean insulin was **63.8** pmol/L | MD **6.43 lower**  (15.08 lower to 2.22 higher) |  | 190  (4 RCTs) | ⊕⊝⊝⊝  VERY LOW ^2,3,5^ |
| **Total cholesterol (mmol/L)**  Follow-up: 2-24 weeks | The mean total cholesterol was  **4.8**mmol/L | MD **0.1 lower**  (0.35 lower to 0.15 higher) |  | 668  (9 RCTs) | ⊕⊕⊝⊝  LOW ^1,2^ |
| **HDL-c (mmol/L)**  Follow-up: 2-24 weeks | The mean c-HDL was  **1.2**mmol/L | MD **0.02 higher**  (0.05 lower to 0.09 higher) |  | 668  (9 RCTs) | ⊕⊕⊝⊝  LOW ^1,2^ |
| **LDL-c (mmol/L)**  Follow-up: 2-24 weeks | The mean c-LDL was  **3.1**mmol/L | MD **0.06 lower**  (0.28 lower to 0.16 higher) |  | 668  (9 RCTs) | ⊕⊕⊝⊝  LOW ^1,2^ |
| **Triglycerides (mmol/L)**  Follow-up: 2-24 weeks | The mean triglyceride level was **1.5**mmol/L | MD **0.13 lower**  (0.2 lower to 0.07 higher) |  | 667  (9 RCTs) | ⊕⊕⊕⊝  MODERATE ^2^ |
| **Total cholesterol/HDL-c**  Follow-up: 3.4-24 weeks | The mean total cholesterol/c-HDL was  **4.7** | MD **0.26 lower**  (0.57 lower to 0.05 higher) |  | 333  (5 RCTs) | ⊕⊝⊝⊝  VERY LOW ^2,3,4^ |
| **LDL-c/HDL-c**  Follow-up: 2-24 weeks | The mean c-LDL/c-HDL was **2.8** | MD **0.14 lower**  (0.34 lower to 0.07 higher) |  | 419  (7 RCTs) | ⊕⊕⊝⊝  LOW ^2,4^ |
| **DBP (mmHg)**  Follow-up: 4-24 weeks | The mean DBP was  **79.1**mmHg | MD **0.5 lower**  (2.44 lower to 1.44 higher) |  | 395  (3 RCTs) | ⊕⊝⊝⊝  VERY LOW ^2,4,5^ |
| **SBP (mmHg)**  Follow-up: 4-24 weeks | The mean SBP was  **119.8** mmHg | MD **0.82 lower**  (3.59 lower to 1.95 higher) |  | 395  (3 RCTs) | ⊕⊝⊝⊝  VERY LOW ^2,4,5^ |
| *****The risk in the peanut group (and its 95% confidence interval) is based on the assumed risk in the comparison group and the relative effect of the intervention (and its 95% CI). BMI: Body mass index; CI: Confidence interval; DBP: Diastolic blood pressure; HDL-c: High-density lipoprotein cholesterol; LDL-c: Low-density lipoprotein cholesterol; MD: Mean difference; RCTs: Randomized controlled trials, SBP: Systolic blood pressure.  ^1^ Heterogeneity of participants  ^2^ Studies involved different participants and comparator groups, as well as different follow-up duration  ^3^ Small sample size (less than 400 participants)  ^4^ Heterogeneity of intervention  ^5^ Bias because of the effect estimate | | | | | |

Randomization

Wash-out period

Baseline assessment

Final assessment

**Supplementary Figure 1.** Flowchart of ARISTOTLE study. SRP: skin roasted peanuts; PB: peanut butter; CB: control butter.

Records identified through database searching (n = 4,100)

PubMed (n = 1,645)

Web of Science (n = 2,007)

Cochrane Library (n = 244)

Scopus (n = 214)

Records duplicated

(n = 970)

Records screened
(n = 3,130)

Included

Screening

Identification

Records excluded after title/abstract screening: reviews/meta-analysis/editorials/ecological studies; in vitro or animal studies; other endpoints/outcomes

(n = 3,101)

Full-text articles assessed for eligibility
(n = 29)

Full-text articles excluded, with reasons

Not randomized clinical trial.

Not controlled

(n = 19)

Eligibility

Studies included in qualitative synthesis
(n = 10)

**ARISTOTLE Study**

Studies included in quantitative synthesis (meta-analysis)
(n = 11)

**Supplementary Figure 2.** PRISMA flow diagram of the study selection for the systematic review and meta-analysis.

| 1. **Body weight**   Curve (estimate (95% CI)): 0.033 (0.000, 0.066), *P*=0.049 | 1. **Body mass index**   Curve (estimate (95% CI)): 0.007 (-0.010, 0.025), *P*=0.406 |
| --- | --- |
| 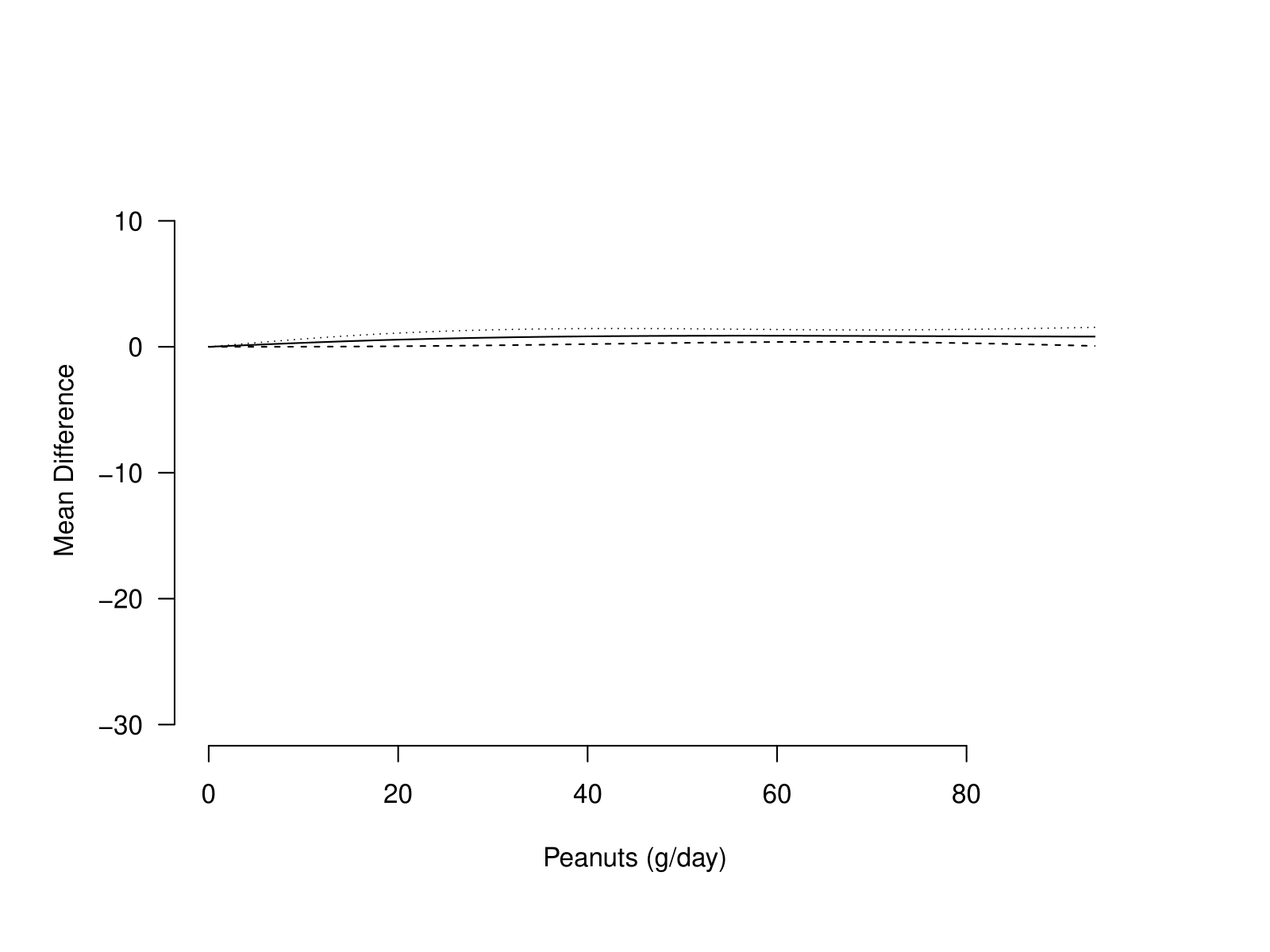 | 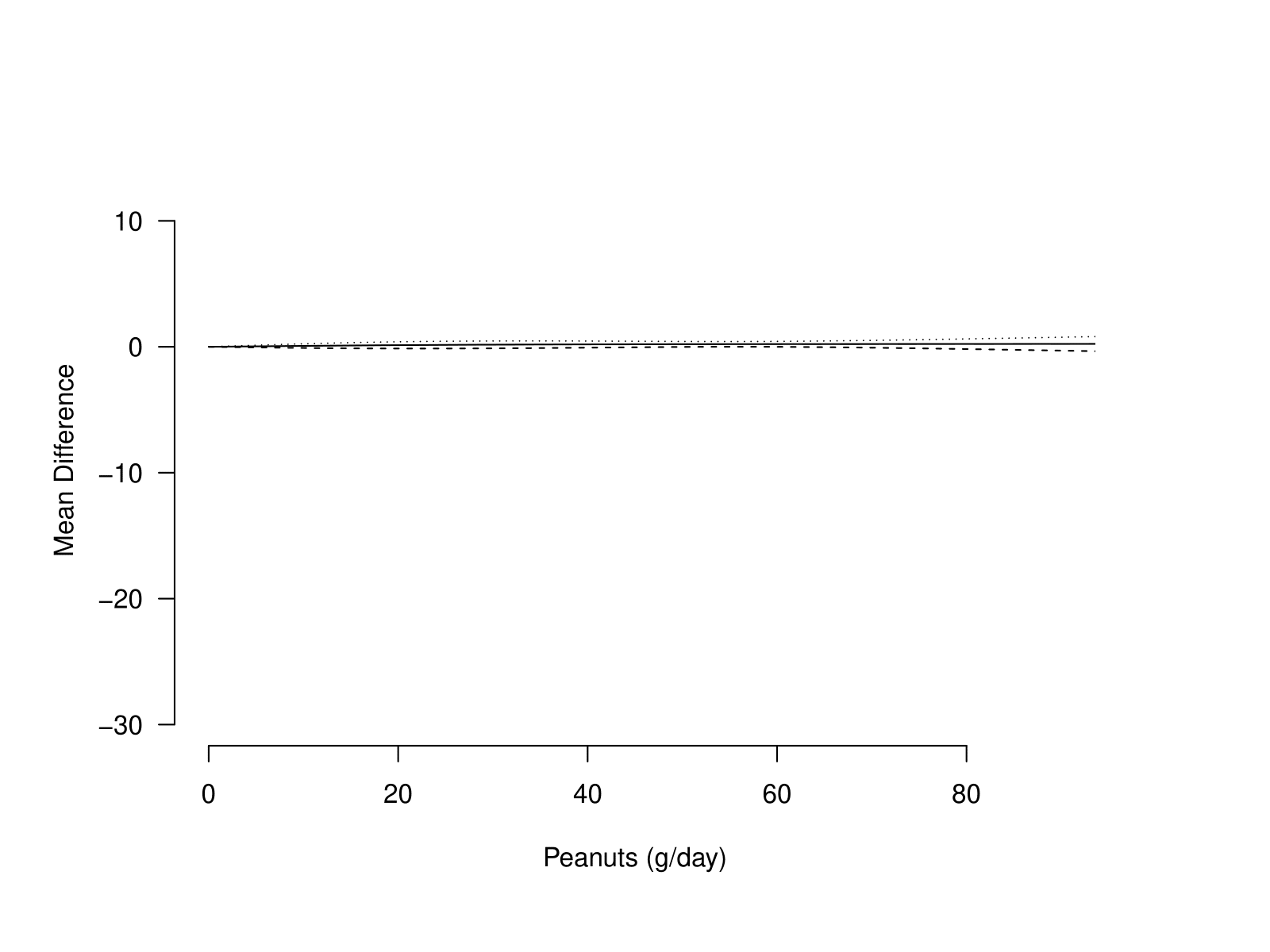 |
| 1. **Body fat**   Curve (estimate (95% CI)): 0.034 (-0.021, 0.089), *P*=0.224 | 1. **Waist circumference**   Curve (estimate (95% CI)): 0.012 (-0.022, 0.043), *P*=0.518 |
| 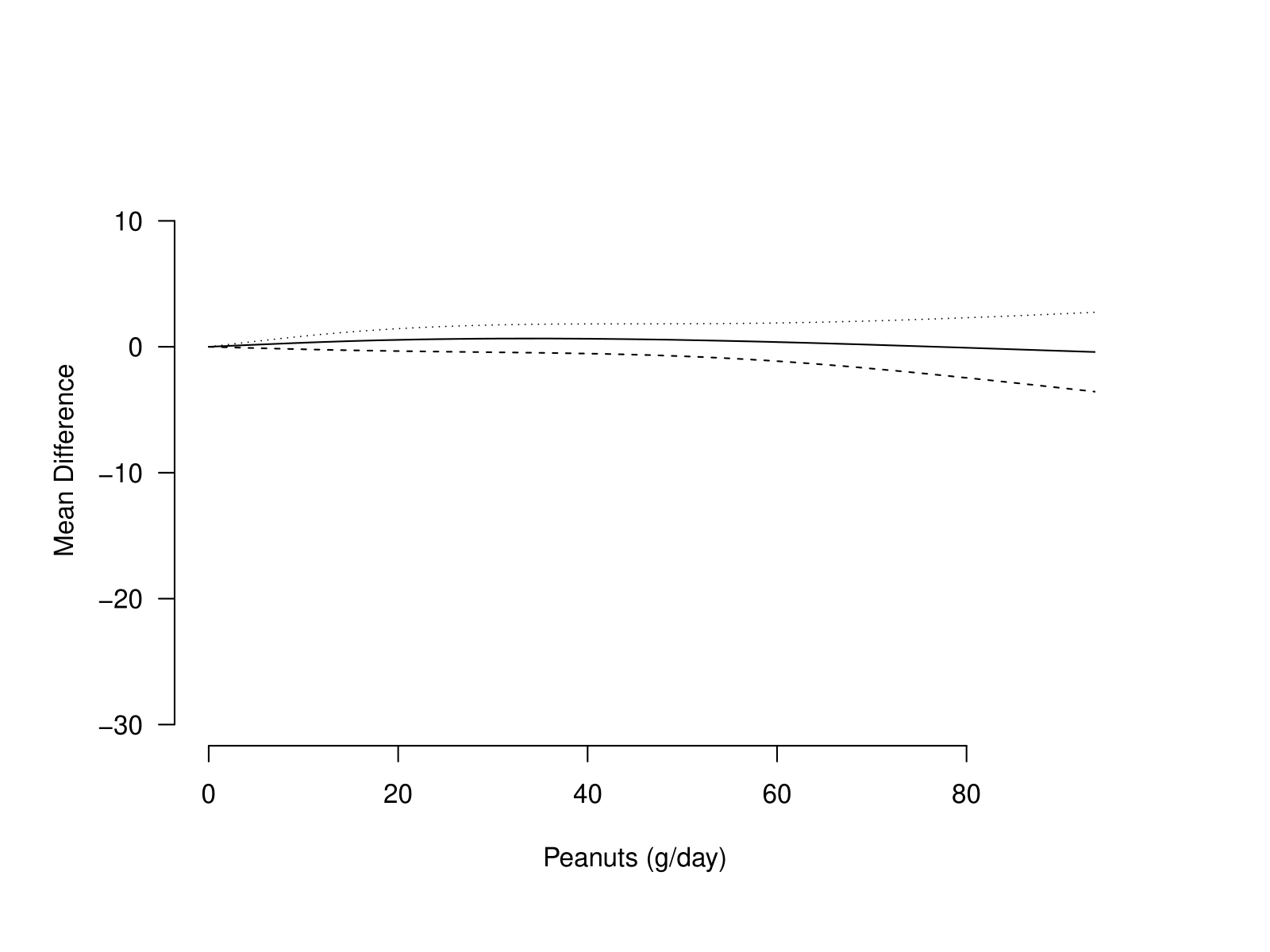 | 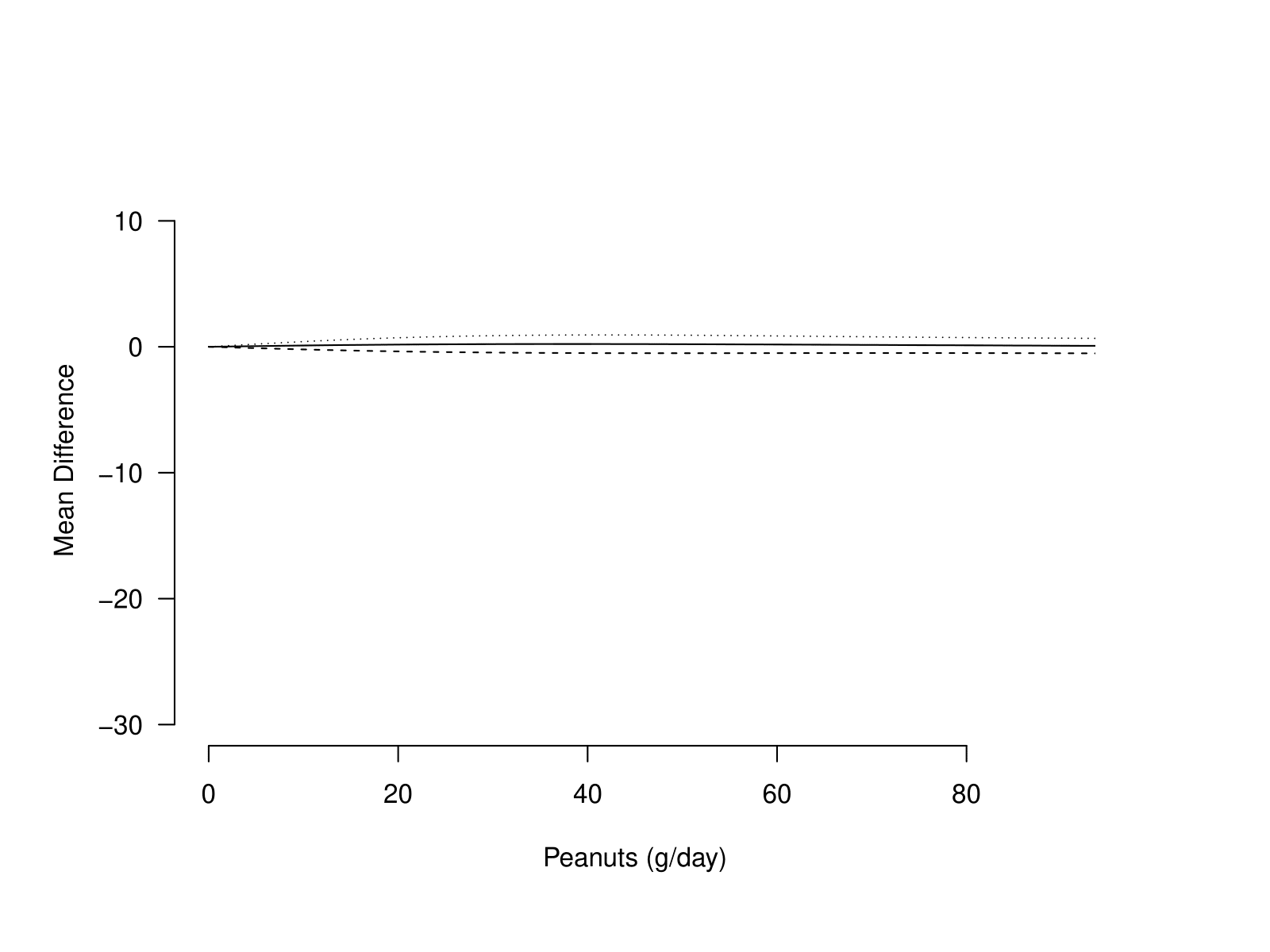 |
| **Supplementary Figure 3. Continuous dose-response meta-analysis of the effects of peanut intake (g/d) vs. control on mean change in anthropometric measurements.**  Pooled dose-response linear associations between peanut intake and mean change in anthropometric measurements (solid line). Peanuts were modeled with restricted cubic splines in a random-effects model. The curve estimates refer to the changes in each anthropometric outcome per each gram increase of peanut intake. The lower 95% CI is represented by a dashed line and the upper 95% CI is represented by a dotted line. These lines represent the 95% confidence intervals for the spline model. | |

| 1. **Glucose**   Curve (estimate (95% CI)): 0.002 (-0.004, 0.008), *P*=0.572 | 1. **Insulin**   Curve (estimate (95% CI)): -0.058 (-0.469, 0.358), *P*=0.784 |
| --- | --- |
| **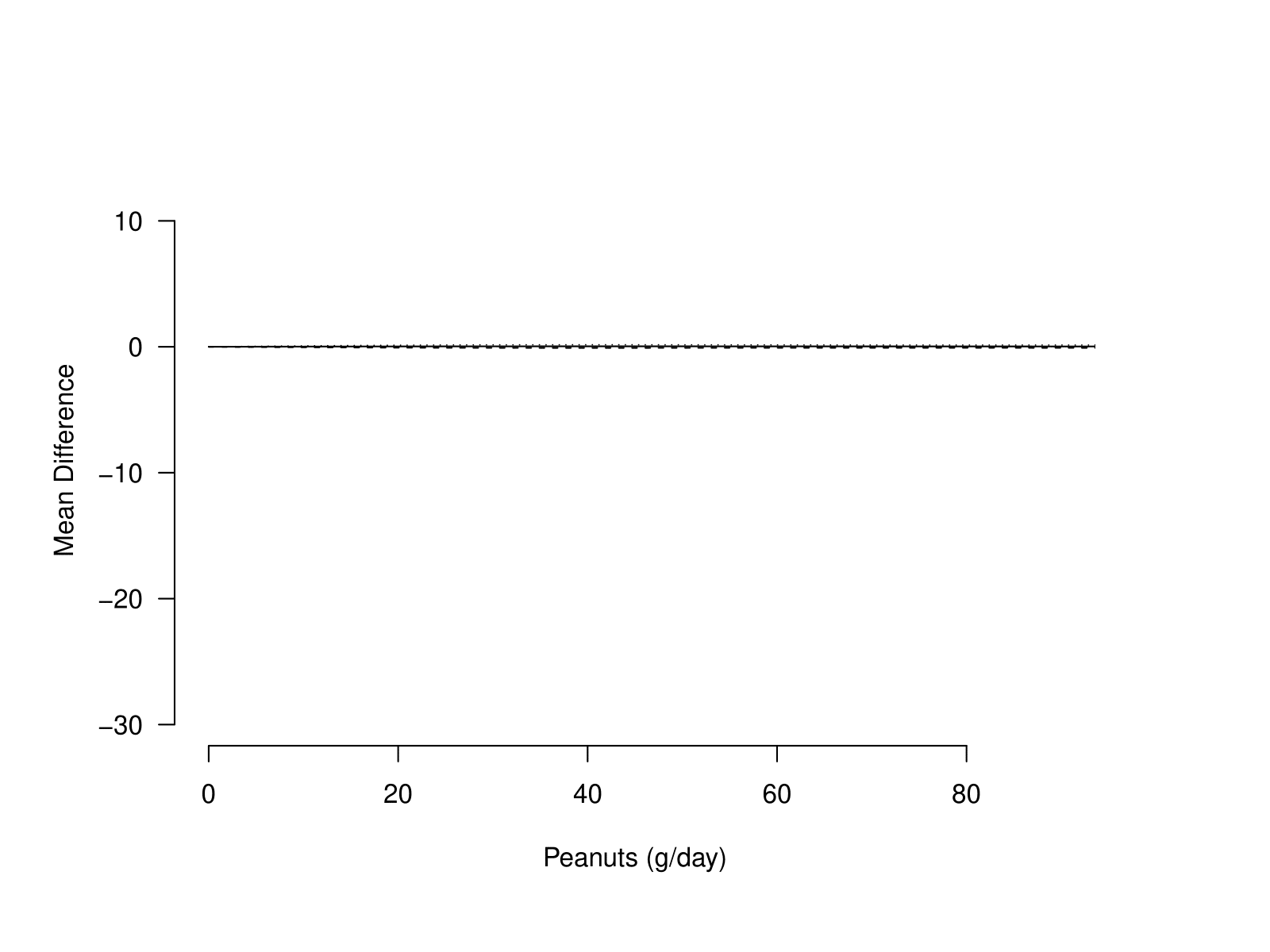** | **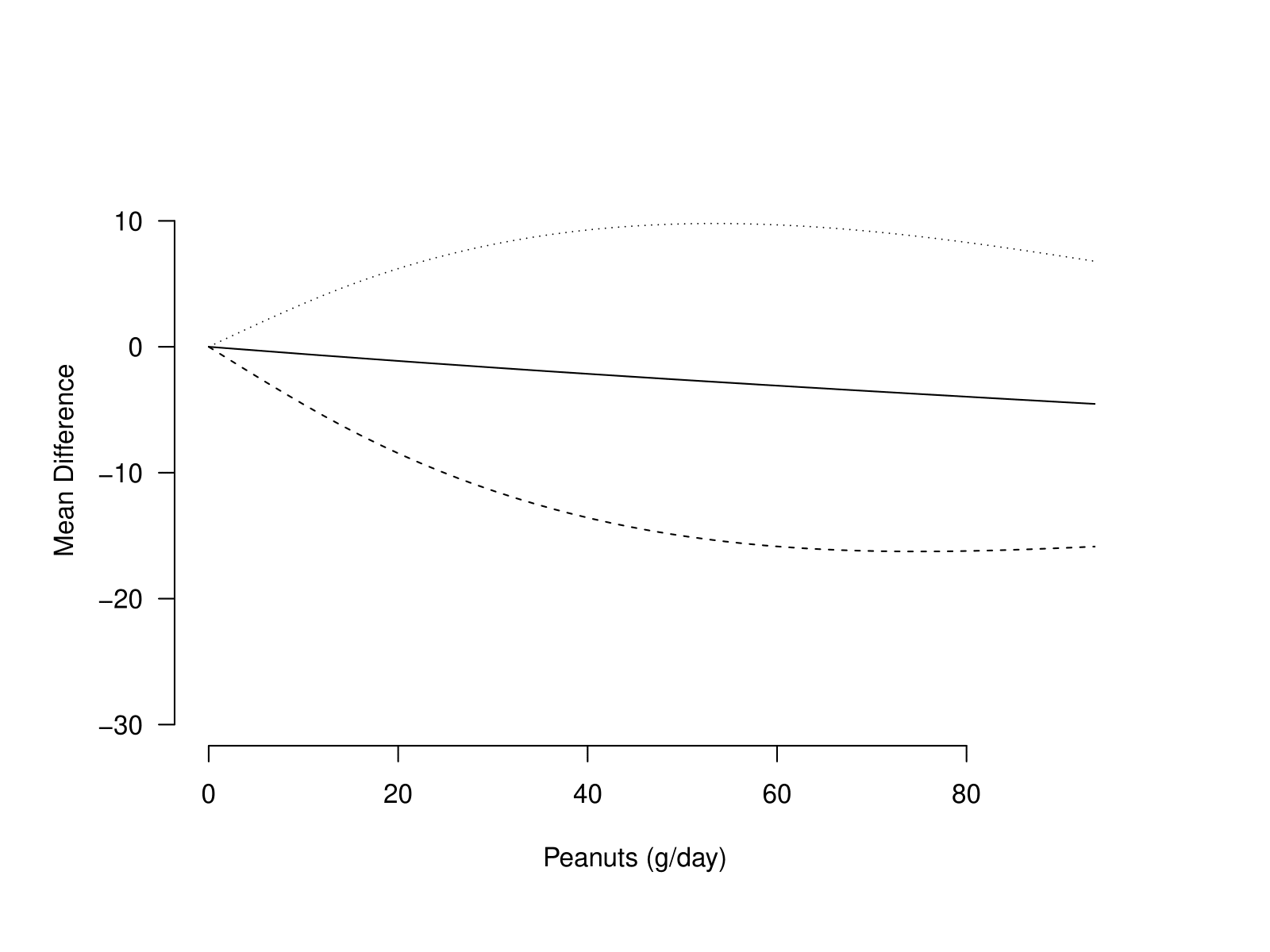** |
| **Supplementary Figure 4. Continuous dose-response meta-analysis of the effects of peanut intake (g/d) vs. control on mean change in glucose metabolism.**  Pooled dose-response linear associations between peanut intake and mean change in glucose or insulin (solid line). Peanuts were modeled with restricted cubic splines in a random-effects model. The curve estimates refer to the changes in glucose and insulin (in mmol/L) per each gram increase of peanut intake. The lower 95% CI is represented by a dashed line and the upper 95% CI is represented by a dotted line. These lines represent the 95% confidence intervals for the spline model. | |

| 1. **Total cholesterol**   Curve (estimate (95% CI)): -0.002 (-0.014, 0.011), *P*=0.802 | 1. **HDL-cholesterol**   Curve (estimate (95% CI)): 0.002 (-0.002, 0.007), *P*=0.276 |
| --- | --- |
| **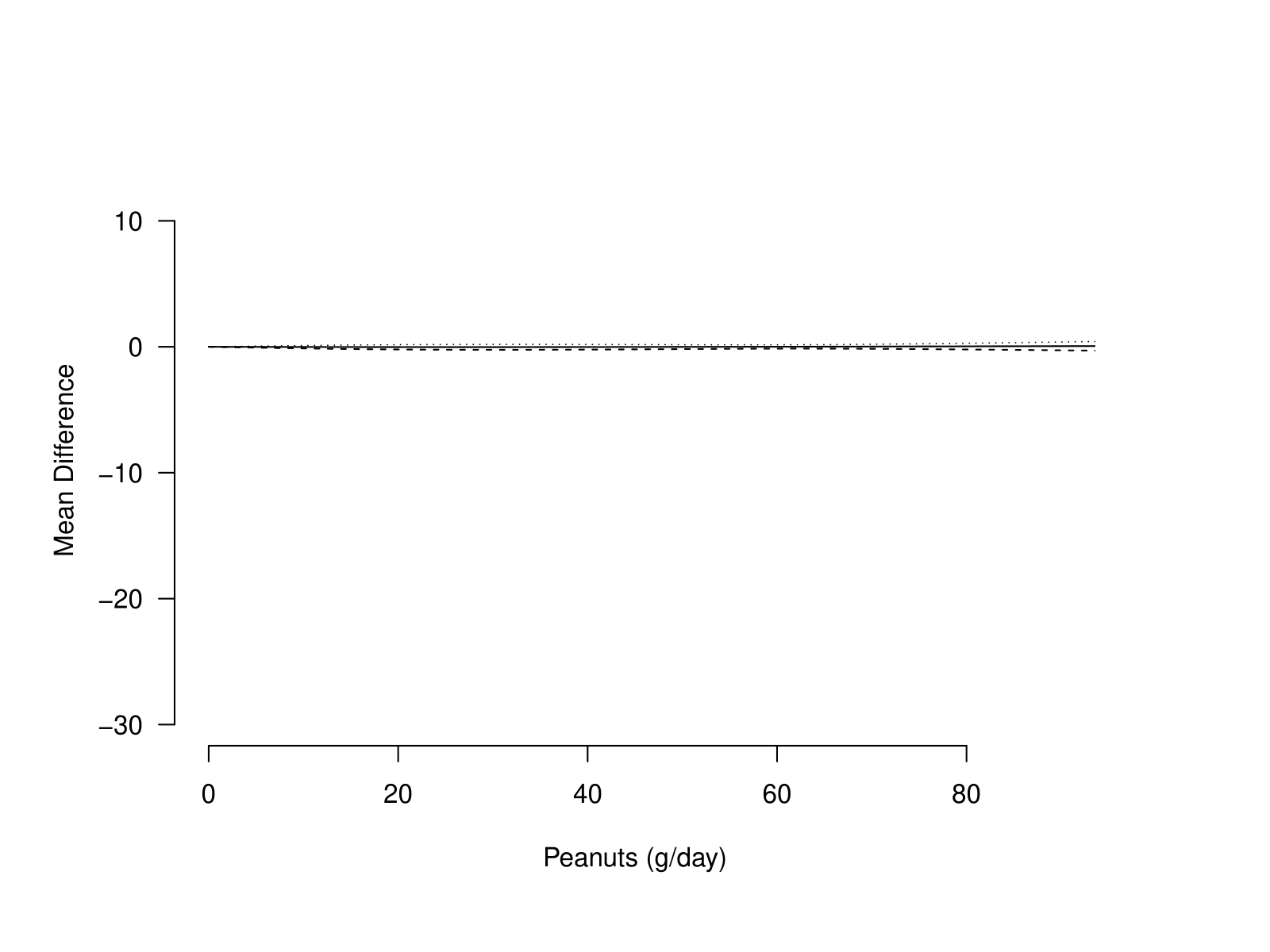** | **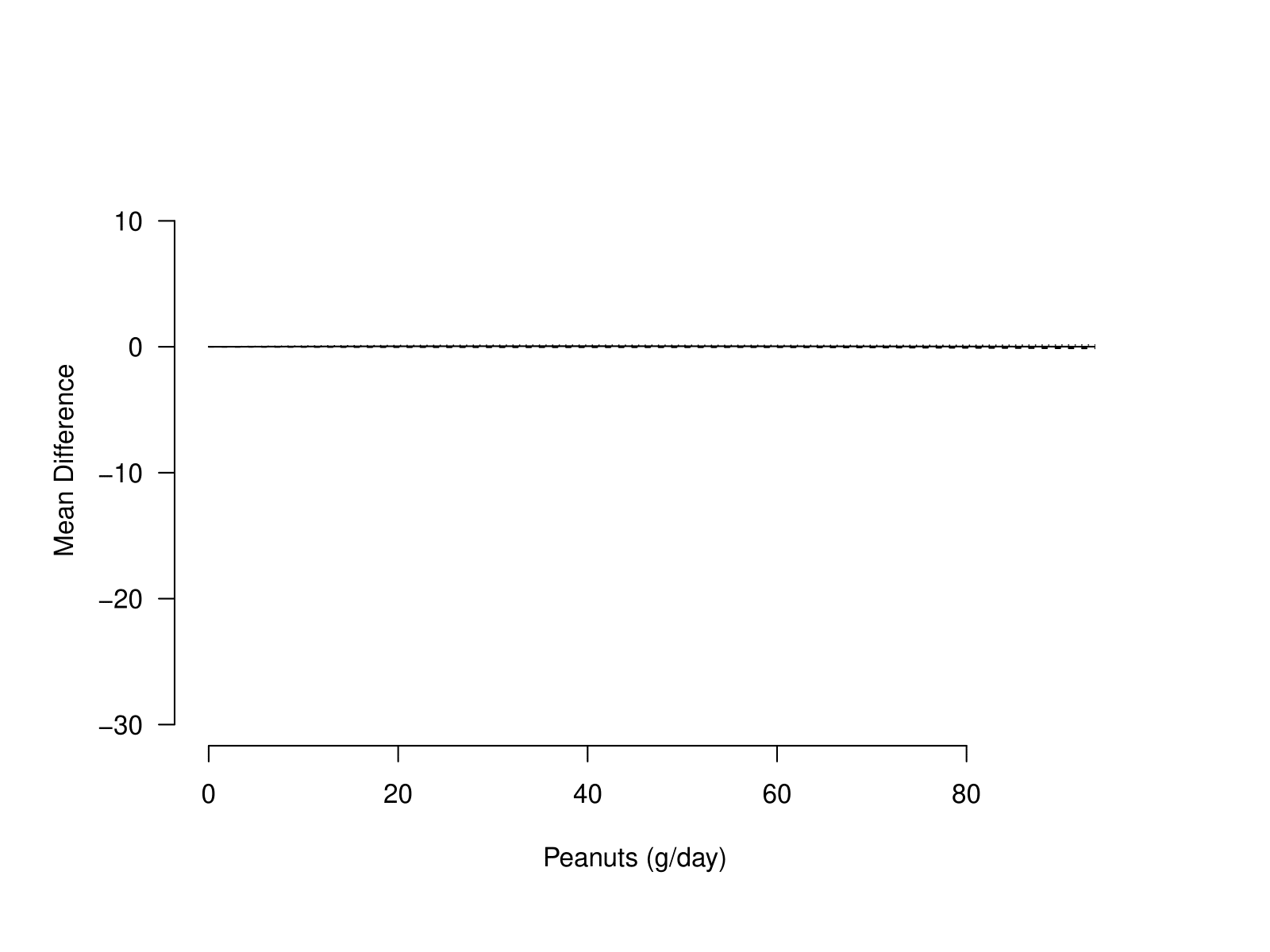** |
| 1. **LDL-cholesterol**   Curve (estimate (95% CI)): 0.004 (-0.002, 0.010), *P*=0.242 | 1. **Triglycerides**   Curve (estimate (95% CI)): -0.001 (-0.006, 0.004), *P*=0.718 |
| **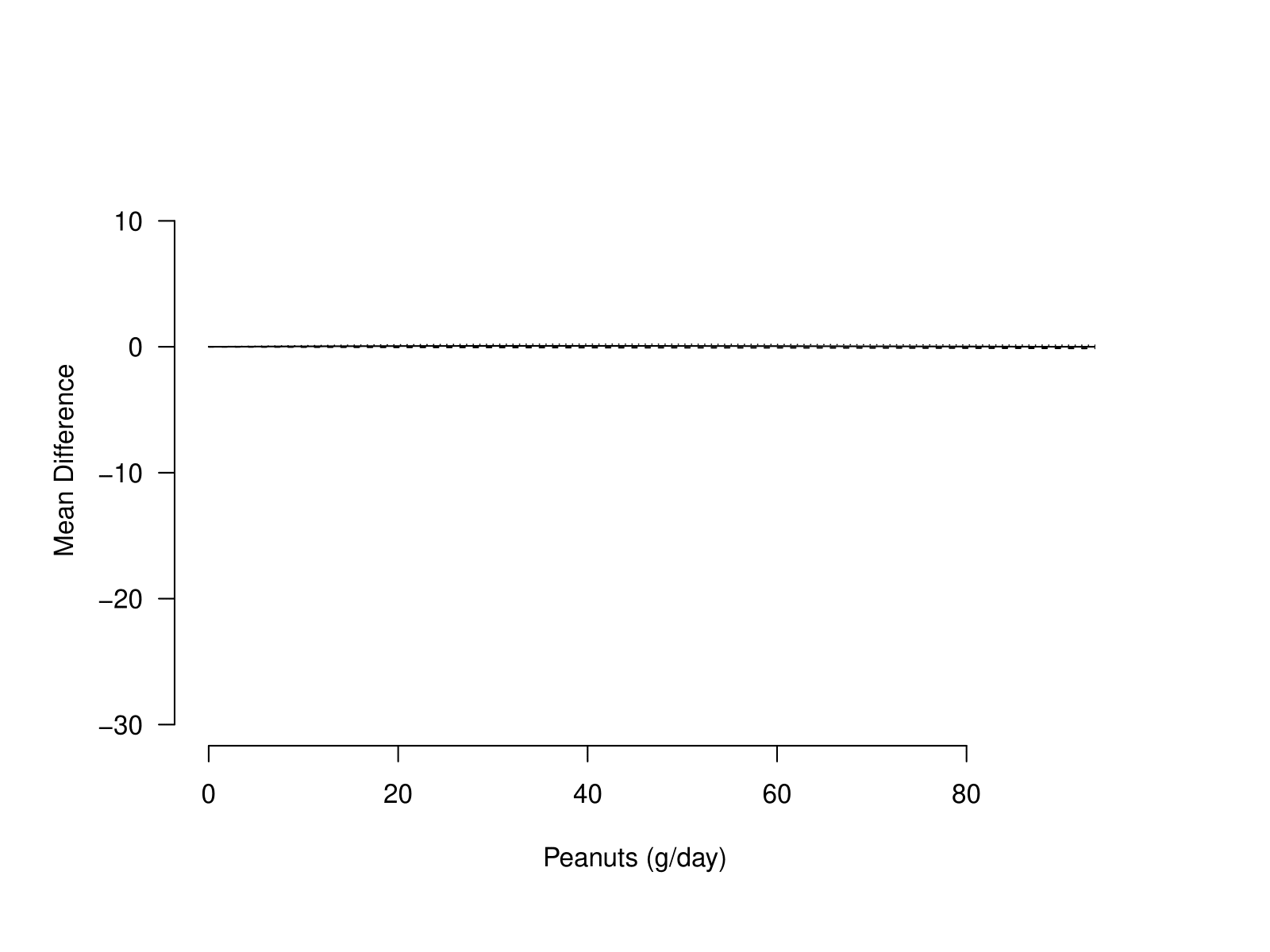** | **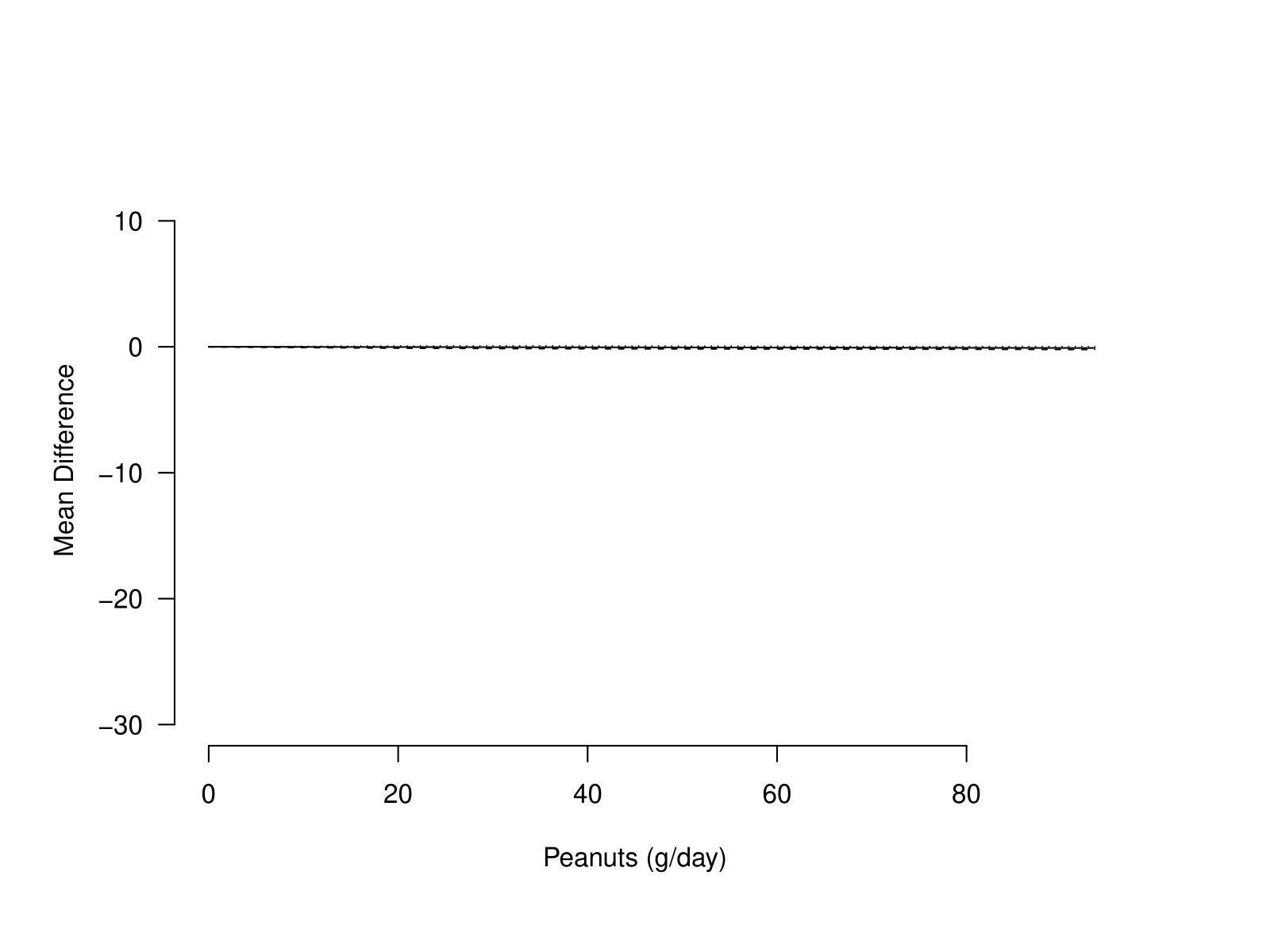** |
| 1. **Total cholesterol/HDL-cholesterol**   Curve (estimate (95% CI)): 0.000 (-0.028, 0.029), *P*=0.981 | 1. **LDL-cholesterol/HDL-cholesterol**   Curve (estimate (95% CI)): -0.002 (-0.013, 0.009), *P*=0.746 |
| **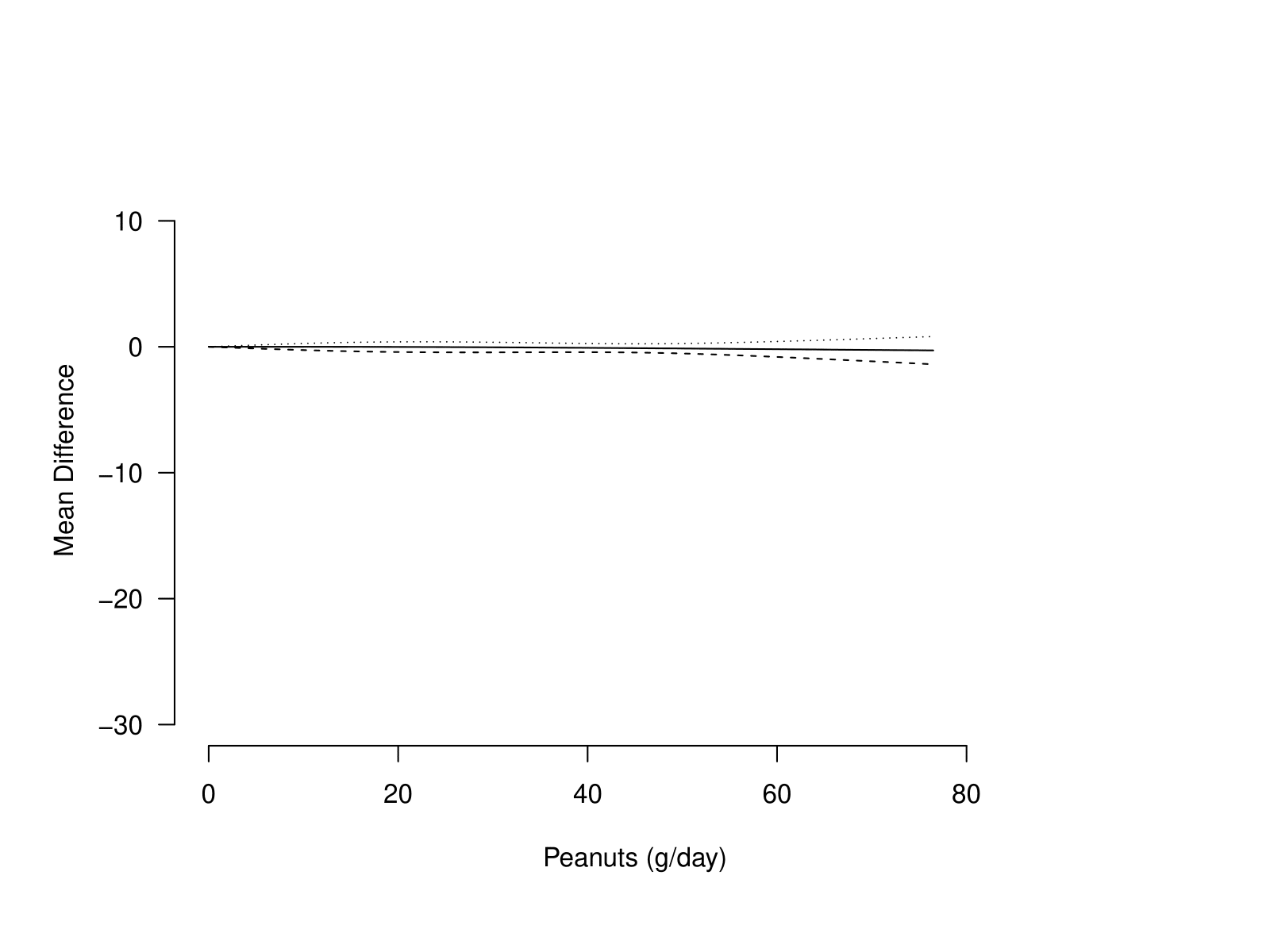** | **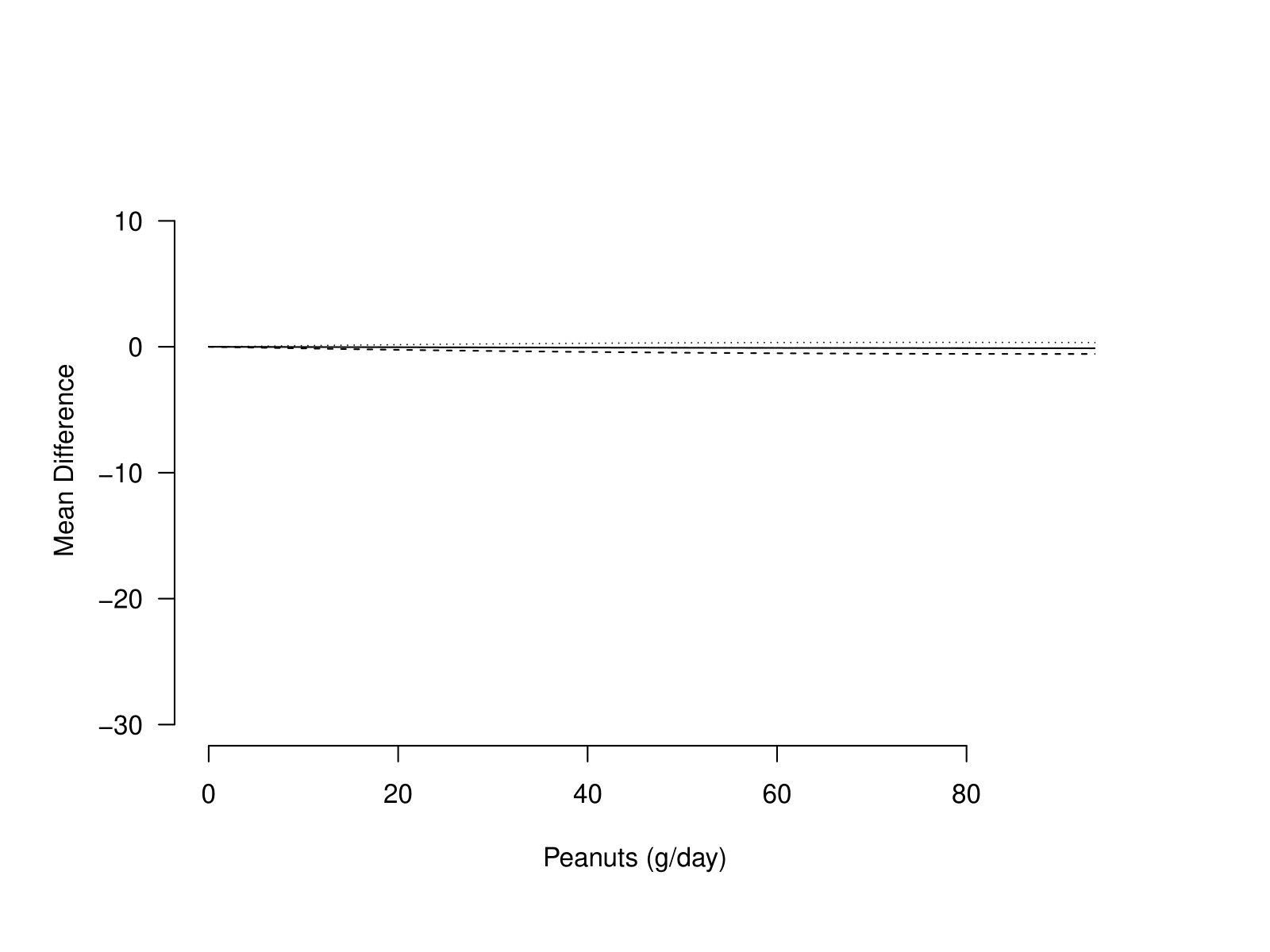** |
| **Supplementary Figure 5. Continuous dose-response meta-analysis of the effects of peanut intake (g/d) vs. control on mean change in blood lipids.**  Pooled dose-response linear associations between peanut intake and mean change in blood lipid concentrations (solid line). Peanuts were modeled with restricted cubic splines in a random-effects model. The curve estimates refer to the changes in blood lipid outcomes (in mmol/L) per each gram increase of peanut intake. The lower 95% CI is represented by a dashed line and the upper 95% CI is represented by a dotted line. These lines represent the 95% confidence intervals for the spline model. | |

| 1. **Systolic blood pressure**   Curve (estimate (95% CI)): 0.107 (-0.110, 0.324), *P*=0.333 | 1. **Diastolic blood pressure**   Curve (estimate (95% CI)): 0.116 (-0.177, 0.410), *P*=0.437 |
| --- | --- |
| **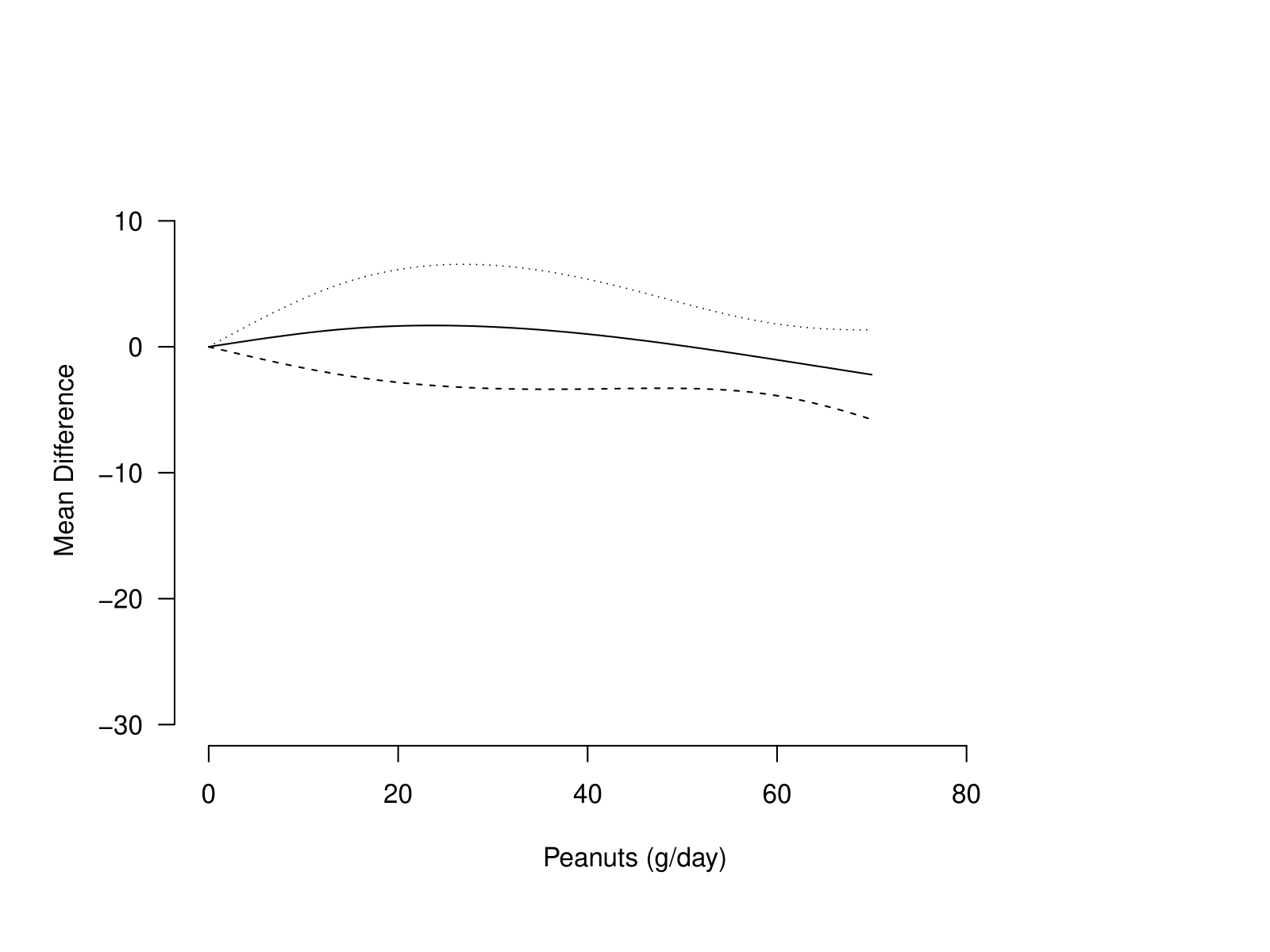** | **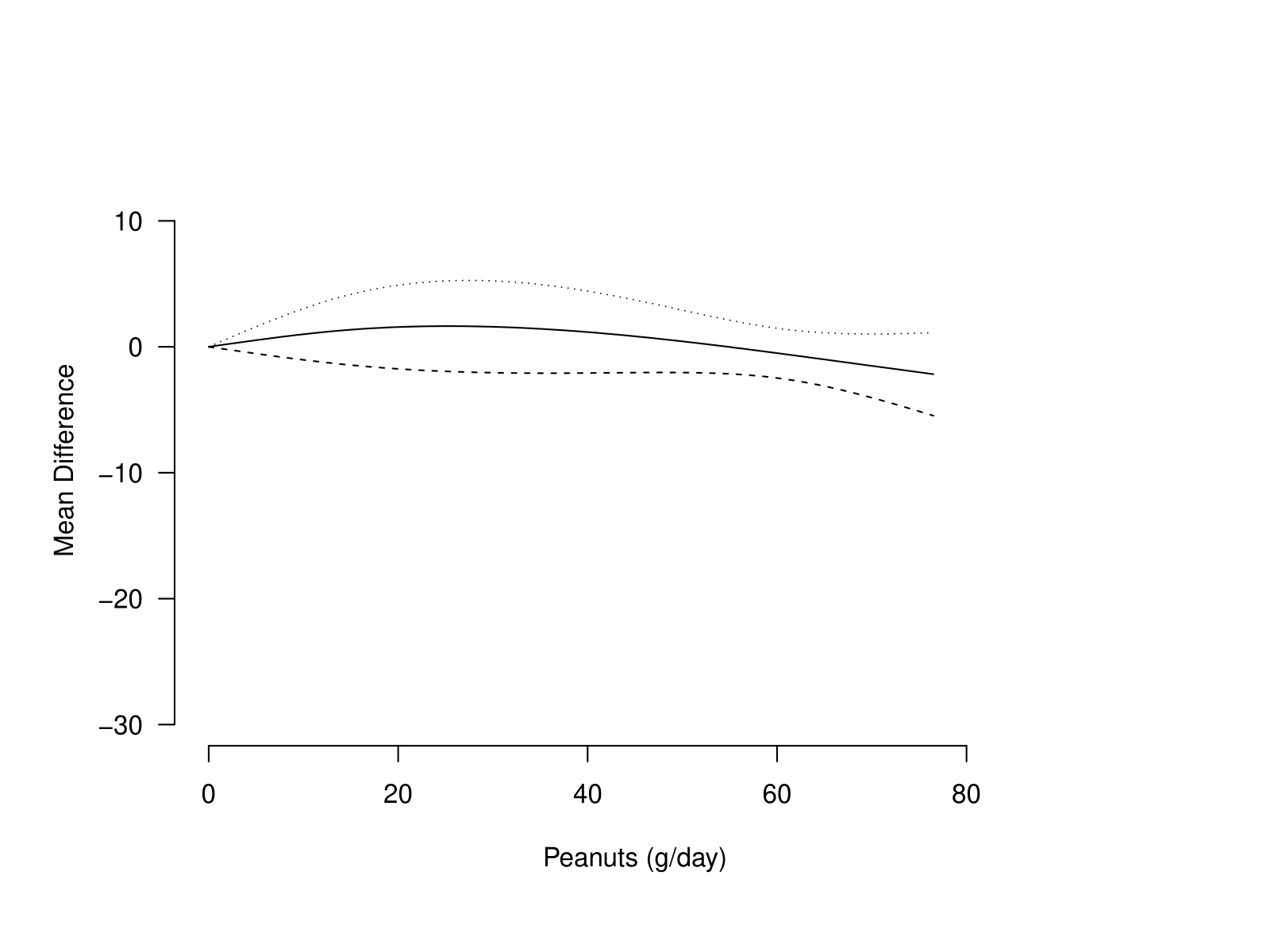** |
| **Supplementary Figure 6. Continuous dose-response meta-analysis of the effects of peanut intake (g/d) vs. control on mean change in blood pressure.**  Pooled dose-response linear associations between peanut intake and mean change in blood pressure (solid line). Peanuts were modeled with restricted cubic splines in a random-effects model. The curve estimates refer to the changes in systolic and diastolic blood pressure (in mmHg) per each gram increase of peanut intake. The lower 95% CI is represented by a dashed line and the upper 95% CI is represented by a dotted line. These lines represent the 95% confidence intervals for the spline model. | |

**Supplementary Figure 7.** Risk of study bias assessed with RoB-2 tool.


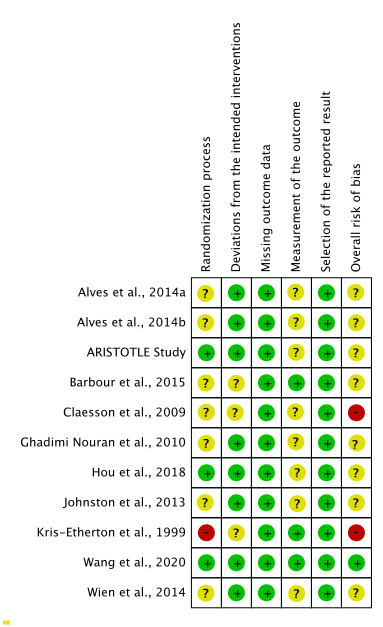

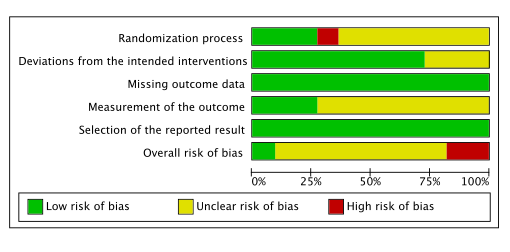


**References**

1. Alper CM, Mattes RD. Effects of chronic peanut consumption on energy balance and hedonics. Int J Obes. 2002;26(8):1129–37.

2. Alper CM, Mattes RD. Peanut consumption improves indices of cardiovascular disease risk in healthy adults. J Am Coll Nutr. 2003;22(2):133–41.

3. Alves RDM, Moreira APB, Macedo VS, Costa NMB, Alfenas R de CG, Bressan J. Cacahuete alto-oleico aumenta la termogénesis inducida por la dieta en hombres con sobrepeso y obesidad. Nutr Hosp. 2014;29(5):1024–32.

4. Barbour JA, Howe PRC, Buckley JD, Bryan J, Coates AM. Cerebrovascular and cognitive benefits of high-oleic peanut consumption in healthy overweight middle-aged adults. Nutr Neurosci. 2017;20(10):555–62.

5. Devitt AA, Kuevi A, Coelho SB, Lartey A, Lokko P, Costa N, et al. Appetitive and dietary effects of consuming an energy-dense food (peanuts) with or between meals by snackers and nonsnackers. J Nutr Metab. 2011;2011.

6. Johnston CS. Strategies for Healthy Weight Loss: From Vitamin C to the Glycemic Response. J Am Coll Nutr. 2005;24(3):158–65.

7. Jones JB, Provost M, Keaver L, Breen C, Ludy MJ, Mattes RD. A randomized trial on the effects of flavorings on the health benefits of daily peanut consumption. Am J Clin Nutr. 2014;99(3):490–6.

8. Lilly LN, Heiss CJ, Maragoudakis SF, Braden KL, Smith SE. The Effect of Added Peanut Butter on the Glycemic Response to a High–Glycemic Index Meal: A Pilot Study. J Am Coll Nutr [Internet]. 2019;38(4):351–7. Available from: https://doi.org/10.1080/07315724.2018.1519404

9. Liu X, Hill AM, West SG, Gabauer RM, McCrea CE, Fleming JA, et al. Acute peanut consumption alters postprandial lipids and vascular responses in healthy overweight or obese men. J Nutr. 2017;147(5):835–40.

10. Lokko P, Lartey A, Armar-Klemesu M, Mattes RD. Regular peanut consumption improves plasma lipid levels in healthy Ghanaians. Int J Food Sci Nutr. 2007;58(3):190–200.

11. McKiernan F, Lokko P, Kuevi A, Sales RL, Costa NMB, Bressan J, et al. Effects of peanut processing on body weight and fasting plasma lipids. Br J Nutr. 2010;104(3):418–26.

12. Moreira APB, Teixeira TFS, Alves RDM, Peluzio MCG, Costa NMB, Bressan J, et al. Effect of a high-fat meal containing conventional or high-oleic peanuts on post-prandial lipopolysaccharide concentrations in overweight/obese men. J Hum Nutr Diet. 2016;29(1):95–104.

13. O’Byrne DJ, Knauft DA, Shireman RB. Low fat-monounsaturated rich diets containing high-oleic peanuts improve serum lipoprotein profiles. Lipids. 1997;32(7):687–95.

14. Reis CEG, Bordalo LA, Rocha ALC, Freitas DMO, da Silva MVL, de Faria VC, et al. Maní tostado y molido conduce a una menor respuesta glicémica postprandial comparado con maní crudo. Nutr Hosp. 2011;26(4):745–51.

15. Reis CEG, Ribeiro DN, Costa NMB, Bressan J, Alfenas RCG, Mattes RD. Acute and second-meal effects of peanuts on glycaemic response and appetite in obese women with high type 2 diabetes risk: A randomised cross-over clinical trial. Br J Nutr. 2013;109(11):2015–23.

16. Shively C, Apgar J, Tarka S. Postprandial glucose and insulin responses to various snacks of equivalent carbohydrate content in normal subjects. Am J Clin Nutr. 1986 Mar;43(3):335–42.

17. Caldas APS, Alves RDM, Hermsdorff HHM, de Oliveira LL, Bressan J. Effects of high-oleic peanuts within a hypoenergetic diet on inflammatory and oxidative status of overweight men: a randomised controlled trial. Br J Nutr. 2020 Mar;123(6):673–80.

18. Tan WSK, Tan WJK, Ponnalagu SDO, Koecher K, Menon R, Tan SY, et al. The glycaemic index and insulinaemic index of commercially available breakfast and snack foods in an Asian population. Br J Nutr. 2018;119(10):1151–6.

19. Traoret CJ, Lokko P, Cruz ACRF, Oliveira CG, Costa NMB, Bressan J, et al. Peanut digestion and energy balance. Int J Obes. 2008;32(2):322–8.

20. Alves RDM, Moreira APB, MacEdo VS, De Cássia Gonçalves Alfenas R, Bressan J, Mattes R, et al. Regular intake of high-oleic peanuts improves fat oxidation and body composition in overweight/obese men pursuing a energy-restricted diet. Obesity. 2014;22(6):1422–9.

21. Moreira Alves RD, Boroni Moreira AP, Macedo VS, Bressan J, De Cássia Gonçalves Alfenas R, Mattes R, et al. High-oleic peanuts: New perspective to attenuate glucose homeostasis disruption and inflammation related obesity. Obesity. 2014;22(9):1981–8.

22. Barbour JA, Howe PRC, Buckley JD, Bryan J, Coates AM. Effect of 12 weeks high oleic peanut consumption on cardio-metabolic risk factors and body composition. Nutrients. 2015;7(9):7381–98.

23. Claesson AL, Holm G, Ernersson Å, Lindström T, Nystrom FH. Two weeks of overfeeding with candy, but not peanuts, increases insulin levels and body weight. Scand J Clin Lab Invest. 2009;69(5):598–605.

24. Ghadimi Nouran M, Kimiagar M, Abadi A, Mirzazadeh M, Harrison G. Peanut consumption and cardiovascular risk. Public Health Nutr. 2010;13(10):1581–6.

25. Hou YY, Ojo O, Wang LL, Wang Q, Jiang Q, Shao XY, et al. A randomized controlled trial to compare the effect of peanuts and almonds on the cardio-metabolic and inflammatory parameters in patients with type 2 diabetes mellitus. Nutrients. 2018;10(11):1–16.

26. Moreno JP, Johnston CA, El-Mubasher AA, Papaioannou MA, Tyler C, Gee M, et al. Peanut consumption in adolescents is associated with improved weight status. Nutr Res [Internet]. 2013;33(7):552–6. Available from: http://dx.doi.org/10.1016/j.nutres.2013.05.005

27. Kris-Etherton PM, Pearson TA, Wan Y, Hargrove RL, Moriarty K, Fishell V, et al. High-monounsaturated fatty acid diets lower both plasma cholesterol and triacylglycerol concentrations. Am J Clin Nutr. 1999;70(6):1009–15.

28. Wang D, Sun L, Liu X, Niu Z, Chen S, Tang L, et al. Replacing white rice bars with peanuts as snacks in the habitual diet improves metabolic syndrome risk among Chinese adults: A randomized controlled trial. Am J Clin Nutr. 2021;113(1):28–35.

29. Wien M, Oda K, Sabaté J. A randomized controlled trial to evaluate the effect of incorporating peanuts into an American Diabetes Association meal plan on the nutrient profile of the total diet and cardiometabolic parameters of adults with type 2 diabetes. Nutr J. 2014;13(1):1–9.
